# Haemagglutination inhibition and virus microneutralisation serology assays: use of harmonised protocols and biological standards in seasonal influenza serology testing and their impact on inter-laboratory variation and assay correlation: A FLUCOP collaborative study

**DOI:** 10.3389/fimmu.2023.1155552

**Published:** 2023-04-18

**Authors:** Joanna Waldock, Edmond J. Remarque, Lingyi Zheng, Sammy Ho, Katja Hoschler, Britta Neumann, Hanna Sediri-Schön, Claudia M. Trombetta, Emanuele Montomoli, Serena Marchi, Giulia Lapini, Fan Zhou, Sarah L. Lartey, Rebecca J. Cox, Marzia Facchini, Maria Rita Castrucci, Damien Friel, Thierry Ollinger, Catherine Caillet, Nedzad Music, Giuseppe Palladino, Othmar G. Engelhardt, the FLUCOP consortium

**Affiliations:** ^1^ Influenza Resource Centre, Vaccines, Science Research & Innovation, Medicines and Healthcare Products Regulatory Agency, Potters Bar, United Kingdom; ^2^ Department of Parasitology, Biomedical Primate Research Centre, Rijswijk, Netherlands; ^3^ Department of Research and Development, Sanofi, Marcy L’Etoile, France; ^4^ Respiratory Viruses Unit, UK Health Secruity Agency, Colindale, United Kingdom; ^5^ Section for Viral Vaccines, Virology Division, Paul-Ehrlich-Institut, Federal Institute for Vaccines and Biomedicines, Langen, Germany; ^6^ Department of Molecular and Developmental Medicine, University of Siena, Siena, Italy; ^7^ Vismederi srl, Siena, Italy; ^8^ Influenza Centre, Department of Clinical Sciences, University of Bergen, Bergen, Norway; ^9^ World Health Organisation (WHO) National Influenza Centre, Department of Infectious, Parasitic and Immune-mediated Diseases, Istituto Superiore di Sanità, Rome, Italy; ^10^ GSK, Wavre, Belgium; ^11^ Seqirus, Cambridge, MA, United States

**Keywords:** influenza viruses, haemagglutination inhibition assay (HAI), virus microneutralisation assay (MN), serology, standardisation

## Abstract

**Introduction:**

The haemagglutination inhibition assay (HAI) and the virus microneutralisation assay (MN) are long-established methods for quantifying antibodies against influenza viruses. Despite their widespread use, both assays require standardisation to improve inter-laboratory agreement in testing. The FLUCOP consortium aims to develop a toolbox of standardised serology assays for seasonal influenza. Building upon previous collaborative studies to harmonise the HAI, in this study the FLUCOP consortium carried out a head-to-head comparison of harmonised HAI and MN protocols to better understand the relationship between HAI and MN titres, and the impact of assay harmonisation and standardisation on inter-laboratory variability and agreement between these methods.

**Methods:**

In this paper, we present two large international collaborative studies testing harmonised HAI and MN protocols across 10 participating laboratories. In the first, we expanded on previously published work, carrying out HAI testing using egg and cell isolated and propagated wild-type (WT) viruses in addition to high-growth reassortants typically used influenza vaccines strains using HAI. In the second we tested two MN protocols: an overnight ELISA-based format and a 3-5 day format, using reassortant viruses and a WT H3N2 cell isolated virus. As serum panels tested in both studies included many overlapping samples, we were able to look at the correlation of HAI and MN titres across different methods and for different influenza subtypes.

**Results:**

We showed that the overnight ELISA and 3-5 day MN formats are not comparable, with titre ratios varying across the dynamic range of the assay. However, the ELISA MN and HAI are comparable, and a conversion factor could possibly be calculated. In both studies, the impact of normalising using a study standard was investigated, and we showed that for almost every strain and assay format tested, normalisation significantly reduced inter-laboratory variation, supporting the continued development of antibody standards for seasonal influenza viruses. Normalisation had no impact on the correlation between overnight ELISA and 3-5 day MN formats.

## 1 Introduction

Seasonal influenza continues to have a high disease burden, infecting an estimated 3-5 million people a year and causing up to 650,000 deaths (WHO seasonal influenza factsheet (1) last accessed 09 January 2023). Annual vaccination is the most effective measure we currently have against infection, reducing both morbidity and mortality. Most licensed vaccines are based upon the immunodominant haemagglutinin (HA) surface protein of the virus and are updated twice yearly as the virus drifts antigenically.

To assess the immunogenicity of current or novel vaccines, to track antigenic drift by serological methods or to define correlates of protection against infection it is critical that we have standardised assays allowing for comparison across testing sites. The two main assays used in serological studies for influenza are the Haemagglutination Inhibition (HAI) assay and the virus MicroNeutralisation (MN) assay. Each has its own advantages and drawbacks. The HAI assay is a quick and inexpensive assay to carry out, requiring little in the way of specialist equipment. There are standardised protocols available to follow, and with recent efforts to harmonise and standardise the assay, inter-laboratory performance is good (2). However, there are some drawbacks to the HAI.

The HAI assay measures a disruption of the interaction between the HA and sialic acid residues on Red Blood Cells (RBCs) – thus only antibodies that bind to the HA receptor binding site or block binding of sialic acid will give an HAI titre. It is known that neutralising antibodies also bind elsewhere on the HA, for example the stem region (3, 4). Thus, some functional antibodies may not be measured using the HAI. In recent years circulating H3N2 viruses have shown greatly reduced binding to avian RBCs (5–8). This loss of avidity has in turn led to changes in the virus neuraminidase (NA) surface protein, resulting in NA-dependent binding of sialic acid residues (6). These viruses typically have low (or no) haemagglutination titres, and must be tested in the presence of an NA inhibitor (e.g. Oseltamivir) in order to measure anti-HA rather than anti-NA antibodies present in sera. They often cannot be characterised by HAI.

MN assays with an ELISA-based readout [or alternatively plaque reduction assays (5, 9)] are a good alternative for antigenic testing of non-agglutinating viruses, as they negate the need for binding to RBCs and are less affected by NA-mediated binding than the HAI (5).

Influenza viruses passaged in eggs (as is typical for seasonal influenza vaccine strains) frequently acquire adaptations that favour growth in eggs over cells. These adaptations can artificially alter the antigenicity of egg passaged viruses [for example through the loss of an N-linked glycosylation site, exposing a highly immunogenic site usually hidden by the glycan (10)], making the egg passaged virus appear antigenically different to cell passaged virus when tested in HAI (11). MN assays seem to be less affected by egg and cell adaptation than the HAI (5) and thus may give a more accurate interpretation of antigenic changes in naturally circulating influenza viruses.

Despite the advantages of MN assays in contrast to HAI they require specialist equipment, are considerably more time consuming and are expensive to run. Additionally, MN assays typically show greater inter-laboratory variability than HAI testing (12–14). Whilst HAI and MN assays both have benefits and drawbacks, it is generally agreed that using both methods for seasonal influenza surveillance gives a good picture of the antigenic drift, with MN data supporting HAI data and providing a clearer picture where issues specific to HAI arise. It is critical, therefore, that we have standardised HAI and MN assays to provide an accurate picture of seasonal virus changes, and for testing of novel vaccination strategies

FLUCOP was a large consortium of 22 members from eight European countries, encompassing academia, vaccine manufacturers, and public health authorities, supported by the Innovative Medicines Initiative Joint Undertaking (IMIJU). The FLUCOP project aimed to develop a toolbox of standardised assays to facilitate the development of existing and novel influenza vaccines, with a breadth of work including traditional influenza serology assays, detection of anti-NA antibodies and cell mediated immunity (15). Building upon numerous studies that demonstrate the positive impact of assay standardisation for HAI/MN serology assays through both assay harmonisation (16) and the use of biological standards (12–14, 17, 18), in this study the FLUCOP consortium presents two large international collaborative studies testing harmonised HAI and MN protocols across 10 participating laboratories. We expanded on previously published work, testing egg and cell isolated and propagated wild-type (WT) viruses in addition to high-growth reassortants typically used as influenza vaccine strains. We tested two MN protocols: an overnight ELISA-based assay and a 3-5 day assay using reassortant viruses and a WT H3N2 cell isolated virus (representing a non-agglutinating strain). We additionally assessed whether biological standards are effective at reducing inter-laboratory variability across serology methods and strains tested.

As serum panels tested in both HAI and MN studies included many overlapping samples, we were additionally able to look at the correlation of HAI and MN titres across different methods and for different influenza subtypes.

## 2 Materials and methods

### 2.1 Antigens

**Table 1** lists all antigens used in this study. Egg propagated viruses were grown in 10-11 day old embryonated hens’ eggs. Cell propagated viruses were grown in MDCK cells (H1N1, B Victoria and B Yamagata strains) or MDCK-SIAT cells (H3N2 strain). Each laboratory was provided with seed viruses for growing in-house stocks of antigen (provided by MHRA), common source antigens for FLUCOP HAI testing (produced at MHRA) and a common stock of receptor destroying enzyme for FLUCOP testing (Denka Seiken, provided by Sanofi).

**Table 1:** Antigens used in testing.

Virus	Isolated and passaged/Antigen type	Collaborative study
IVR-180 (H1N1)pdm09	Egg/HGR	HAI/MN
X-263B (H3N2)	Egg/HGR	HAI/MN
BX-35 (B Victoria)	Egg/HGR	HAI/MN
BX-59A (B Yamagata)	Egg/HGR	HAI/MN
A/Michigan/45/2015 (H1N1)	Egg/WT	HAI
A/Hong Kong/4801/2014 (H3N2)	Egg/WT	HAI
B/Brisbane/60/2008 (B Victoria)	Egg/WT	HAI
B/Phuket/3073/2013 (B Yamagata)	Egg/WT	HAI
A/Michigan/45/2014 (H1N1)	Cell/WT	HAI
A/Yamaguchi/34/2016 (H3N2)	Cell/WT	HAI/MN
B/Texas/02/2013 (B Victoria)	Cell/WT	HAI
B/Phuket/3073/2013 (B Yamagata)	Cell/WT	HAI

HGR, High Growth Reassortant; WT, wild type; HAI, haemagglutination inhibition assay; MN, microneutralisation assay.

### 2.2 Clinical serum samples

Two serum panels were used in this study: Panel 1 for HAI testing and Panel 2 for MN testing. There were 28 overlapping samples between panel 1 and 2. Panel 1 consisted of 30 pre- and post-vaccination human serum samples (University of Ghent: FLUCOP QIV clinical trial, Fluarix Tetra vaccine from 2017-18 containing the following influenza strains: A/Michigan/45/2015 (H1N1)pdm09, A/Hong Kong/4801/2014 (H3N2), B/Brisbane/60/2008 and B/Phuket/3073/2013), 4 post-vaccination human serum samples (Provided by Sanofi, 2015-2016 trivalent influenza vaccine (TIV) (A/California/07/2009, A/South Australia/55/2014, B/Phuket/3073/2013), the 2^nd^ international standard for antibody to A/California/07/2009 like virus (NIBSC code 10/202, a human plasma pool taken from recipients of A/California/07/2009 NYMX X179A vaccine), strain specific ferret sera (provided by MHRA – one monovalent ferret serum for each influenza A subtype and B lineage tested) and three study standards (GhPool1 and 2 – pool of four donors from the FLUCOP QIV clinical trial, Pool 3b – pool of sera from donors vaccinated with the TIV from 2015-16).

Panel 2 consisted of 30 pre- and post-vaccination human samples (University of Ghent: Flucop_QIV clinical trial, Fluarix Tetra vaccine from 2017-18 containing the following influenza strains: A/Michigan/45/2015 (H1N1)pdm09, A/Hong Kong/4801/2014 (H3N2), B/Brisbane/60/2008 and B/Phuket/3073/2013), 4 post-vaccination human serum samples (Provided by Sanofi, 2015-2016 trivalent influenza vaccine (TIV) (A/California/07/2009, A/South Australia/55/2014, B/Phuket/3073/2013), a polyclonal rabbit anti-NA N1 serum (provided by Sanofi) and three study standards (GhPool1 and 2 – pool of four donors from the FLUCOP QIV clinical trial, Pool 3b – pool of sera from donors vaccinated with the TIV from 2015-16).

### 2.3 Participating laboratories for HAI and MN studies

Ten laboratories participated in the HAI collaborative study: GSK, Instituto Superiore di Sanita (ISS), Medicines and Healthcare products Regulatory Agency (MHRA), Paul Ehrlich Institute (PEI), Seqirus, Sanofi, UK Health Security Agency (UKHSA), University of Bergen (UIB), University of Siena (UNISI), Vismederi (alphabetical order and not the order of numbered labs in the study).

For the MN collaborative study: seven laboratories performed the overnight ELISA-based MN assay: ISS, PEI, Seqirus, Sanofi, UKHSA, UNISI, Vismederi; three laboratories performed the FLUCOP 3-5 day MN assay: GSK, Seqirus, Vismederi (alphabetical order and not the order of numbered labs in the study).

### 2.4 FLUCOP harmonised protocol for HAI

A detailed version of the HAI protocol can be found in the previous FLUCOP collaborative publication (2) along with a link to a training video.

### 2.5 FLUCOP harmonised protocols for MN

The overnight ELISA protocol used in this study was the World Health Organisation (WHO) protocol: Serological diagnosis of influenza by microneutralisation assay (https://www.who.int/publications/i/item/serological-diagnosis-of-influenza-by-microneutralization-assay, accessed 16 March 2023 (19)).

Laboratories performed the ELISA readout of this protocol using 3, 3’, 5, 5’-Tetramethylbenzidine (TMB) substrate due to the restricted use of o-phenylenediamine dihydrochloride (OPD) in some participating laboratories.

The FLUCOP 3-5 day MN protocol used in this study can be found in the **Supplementary Materials**. For this method haemagglutination (HA)/cytopathic effect (CPE) and ELISA readouts could be used according to the laboratory’s preference.

### 2.6 Statistical analysis

Data were read and decoded from the Excel report workbooks supplied by the participating laboratories. Titres < 10 were assigned a value of 5 for calculations and titres of duplicate samples were combined into one geometric mean titre (GMT). Data were expressed as the reciprocal of serum dilutions. The between laboratory variability in titres was calculated per sample across laboratories with available data and was expressed as the standard deviation of the log2-transformed titres.

Titres were normalised by calculating a calibration factor per run (the ratio of the serum standard titre in a run/the global GMT of the serum standard across all testing laboratories). The calibration factor was then applied to all other titres within that run.

For the HAI assays, serum locations in the panel were randomised and replicates were tested in two separate runs, with the operators blinded for the location of the replicates.

For the MN, sera were not randomised, with duplicate samples assayed in the same run. Sera were re-tested when duplicate titres differed by more than 4-fold (samples were re-tested no more than twice). Two independent runs were carried out for each MN assay format.

## 3 Results

### 3.1 HAI data returned

10 laboratories participated in the study. Laboratories were asked to test the 4 representative seasonal strains in the following format: High Growth Reassortant (HGR) seasonal viruses, egg grown wild-type (WT) viruses antigenically identical to the HGR strains and cell grown WT viruses antigenically similar to the HGR strains (see **Table 1** for antigen details). Laboratories were asked to carry out two repeated runs using a common source antigen (provided to laboratories) with the FLUCOP SOP (FLUCOP testing), and two repeated runs using in-house antigens and in-house protocols (in-house testing). For FLUCOP testing, 10 laboratories returned data. For in-house testing, 8 laboratories returned data. Five laboratories returned data for testing H3N2 WT egg propagated virus (due to difficulties with non-agglutination of avian RBCs). Due to errors during runs, only seven laboratories returned data for H1N1 cell WT and B Victoria Egg WT testing. The FLUCOP HAI SOP recommends re-testing of sera if certain quality criteria are not met, however in this study participants were not able to re-test due to limitations on the volume of sera samples required for such a large collaborative study. Comparisons between FLUCOP vs in-house testing were carried out using the data returned from the 8 laboratories providing both FLUCOP and in-house results.

#### 3.1.1 Impact of antigen type and FLUCOP testing on HAI titres

**Figures S1/S2** shows the GMTs across the sample panel tested for each virus, antigen type and protocol by lab. **Table 2** gives the overall GMT across the 10 testing labs and all serum samples for each virus, antigen type and using both FLUCOP testing and in-house testing protocols. When looking at FLUCOP testing only (which removed compounding factors such as the mixed use of ether split and native B antigens for in-house testing) H1N1 GMTs for egg grown reassortant and WT viruses were similar, however GMTs for cell grown H1N1 virus were higher. For H3N2 viruses, egg grown reassortant and WT viruses had similar GMTs, but cell grown WT virus gave reduced GMTs. Antigen type had little impact on B Victoria GMTs, but for B Yamagata, egg grown reassortant virus gave lower GMTs than both WT viruses (which were similar). For both A and B viruses a similar pattern was observed in the in-house data set (see **Supplementary Figure S1**), although the titres for B viruses were much lower.

**Table 2:** Overall Geometric Mean HAI Titres (GMTs) for each antigen type.

	H1N1	H3N2	B Vic	B Yam
FLUCOP protocol				
**Reassortant egg**	138.4 (114.4 - 167.3)	138.2 (115.9 - 164.8)	359.3 (297.2 - 434.5)	203.0 (171.1 - 240.9)
**WT egg**	159.4 (131.9 - 192.6)	99.5 (79.3 - 124.8)	351.3 (290.8 - 424.3)	311.2 (264.3 - 366.3)
**WT cell**	252.4 (203.8 - 312.6)	58.5 (48.8 - 70.2)	295.3 (251.0 - 347.5)	292.3 (246.8 - 346.3)
In-House protocol				
**Reassortant egg**	153.2 (124.3 - 188.9)	153.2 (125.9 - 186.5)	77.6 (63.3 - 95.2)	41.5 (34.8 - 49.4)
**WT egg**	199.2 (161.6 - 245.5)	109.7 (86.5 - 139.2)	50.5 (40.9 - 62.4)	58.5 (49.0 - 70.0)
**WT cell**	267.5 (209.3 - 342.0)	73.7 (60.9 - 89.2)	68.4 (56.4 - 83.0)	74.0 (61.8 - 88.6)

Overall GMTs across the serum panel and all testing labs for each antigen type are shown (GMT(range of titres)).

**Figure 1:**
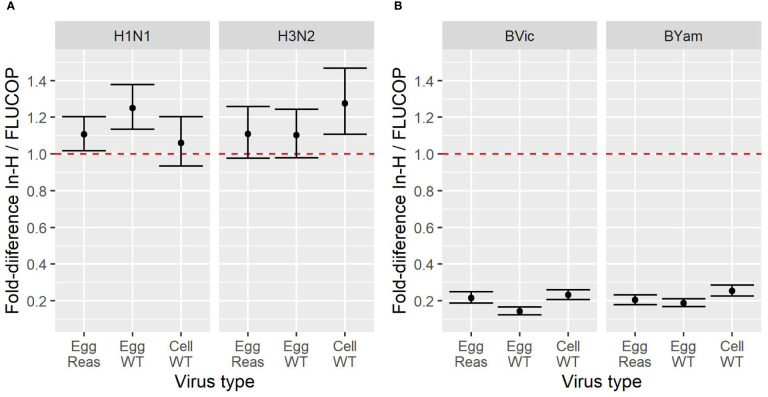
Effect of antigen type on GMT for in-house and FLUCOP HAI testing for **[1A]** A viruses and **[1B]** B viruses. A shared human serum panel was tested in 10 laboratories with three antigenically similar viruses from each seasonal influenza subtype/lineage –egg propagated reassortant viruses (Egg Reas), egg isolated and propagated WT viruses (Egg WT) and cell isolated and propagated WT viruses (Cell WT). Viruses were tested by HAI using in-house testing (In-H) with in-house antigens or using a FLUCOP consensus protocol and common source viruses (FLUCOP). The difference is represented as the fold difference in GMT of in-house/FLUCOP testing. The red dashed line indicates a fold change of 1 i.e. where in-house and FLUCOP testing gave the same GMT. Fold differences higher than 1 indicate titres are higher using in-house testing, and fold differences of less than 1 indicate titres are lower using in-house testing. Overall GMTs across the serum panel for each condition and laboratory are shown in **figure S1**.

To compare the impact of antigen type and protocol type, the fold difference in GMTs between in-house and FLUCOP testing were calculated for each antigen type. **Figure 1** shows the impact on fold difference in in-house/FLUCOP GMTs when testing our panel of viruses for H1N1, H3N2, B Victoria and B Yamagata subtype/lineage viruses. We see a clear difference between A and B viruses: titres for in-house testing for A viruses were almost always higher than FLUCOP testing, however for the B viruses, in-house testing gave much lower titres – this is likely due to the use of ether split antigen for FLUCOP testing (but mixed use of native and ether split antigen for in-house testing) as demonstrated in previous studies (20, 21).

#### 3.1.2 Impact of antigen type and FLUCOP testing on inter-laboratory agreement in HAI testing

**Figure 2:**
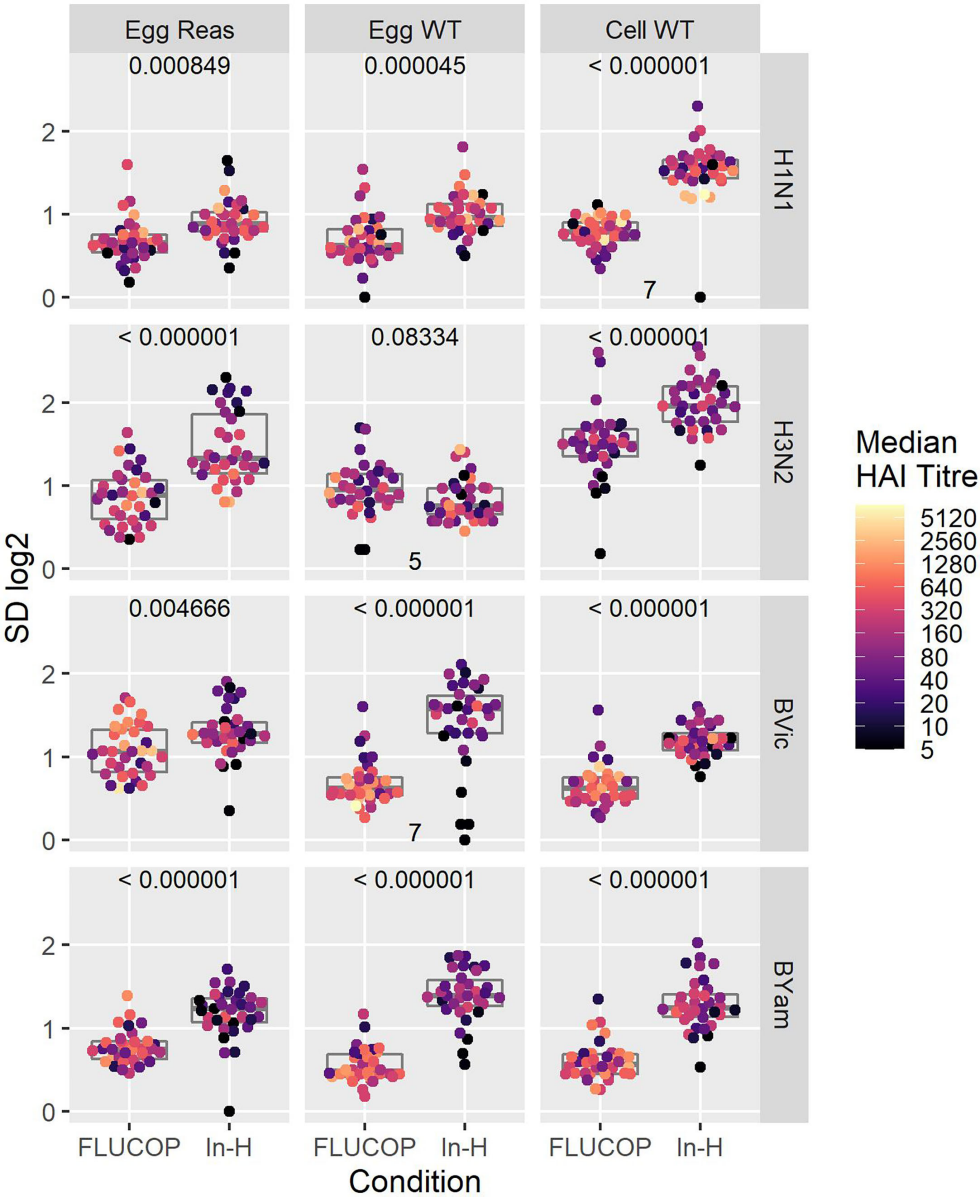
Effect of harmonisation on interlaboratory variation. A shared serum panel was tested by 8 laboratories using egg propagated reassortant antigens (Egg Reas), egg isolated and propagated WT antigens (Egg WT) and cell isolated and propagated antigens (Cell WT) for each seasonal influenza A subtype and B lineage. Standard deviations (SD) are plotted for each sample, with median HAI/sample shaded by titre. P values (comparison of SDs when testing with the FLUCOP protocol and common source antigens (FLUCOP) or with in-house protocols and in-house antigens (In-H)) using paired T tests are shown at the top of each panel. Where fewer than 8 laboratories returned data the number of sets included in the analysis is indicated at the bottom of the panel.

We went on to the assess the impact of antigen type and protocol on inter-laboratory variation. **Figure 2** shows the SD per sample for FLUCOP and in-house testing for egg grown reassortant viruses, egg grown WT viruses and cell grown WT viruses. When comparing FLUCOP and in-house testing across our panel of viruses, for almost all (11/12) subtypes/lineages and antigen types, FLUCOP testing resulted in significantly lower SD than in-house testing (see **Figure 2**). The single exception was H3N2 WT egg propagated antigen. Here, there is no difference between the two testing methods, however only 5 of the 8 laboratories returned data for this antigen due to difficulties with non-agglutination of avian RBCs. For H3N2 both egg propagated reassortant and cell propagated WT viruses showed lower SD with FLUCOP testing. For in-house testing of the A virus strains, cell propagated WT virus showed higher inter-laboratory variation than egg propagated reassortant and WT viruses. FLUCOP testing followed a similar pattern, with cell viruses giving higher SD (albeit with overall lower variability for almost all A H1N1 and H3N2 antigen types). In-house testing of B strains showed relatively high levels of inter-laboratory variability for egg (WT/reassortant) and cell viruses. FLUCOP testing reduced variability, with broadly similar SDs for all B influenza antigen types.

#### 3.1.3 Impact of normalisation using a study standard on inter-laboratory variability in HAI testing

**Figure 3:**
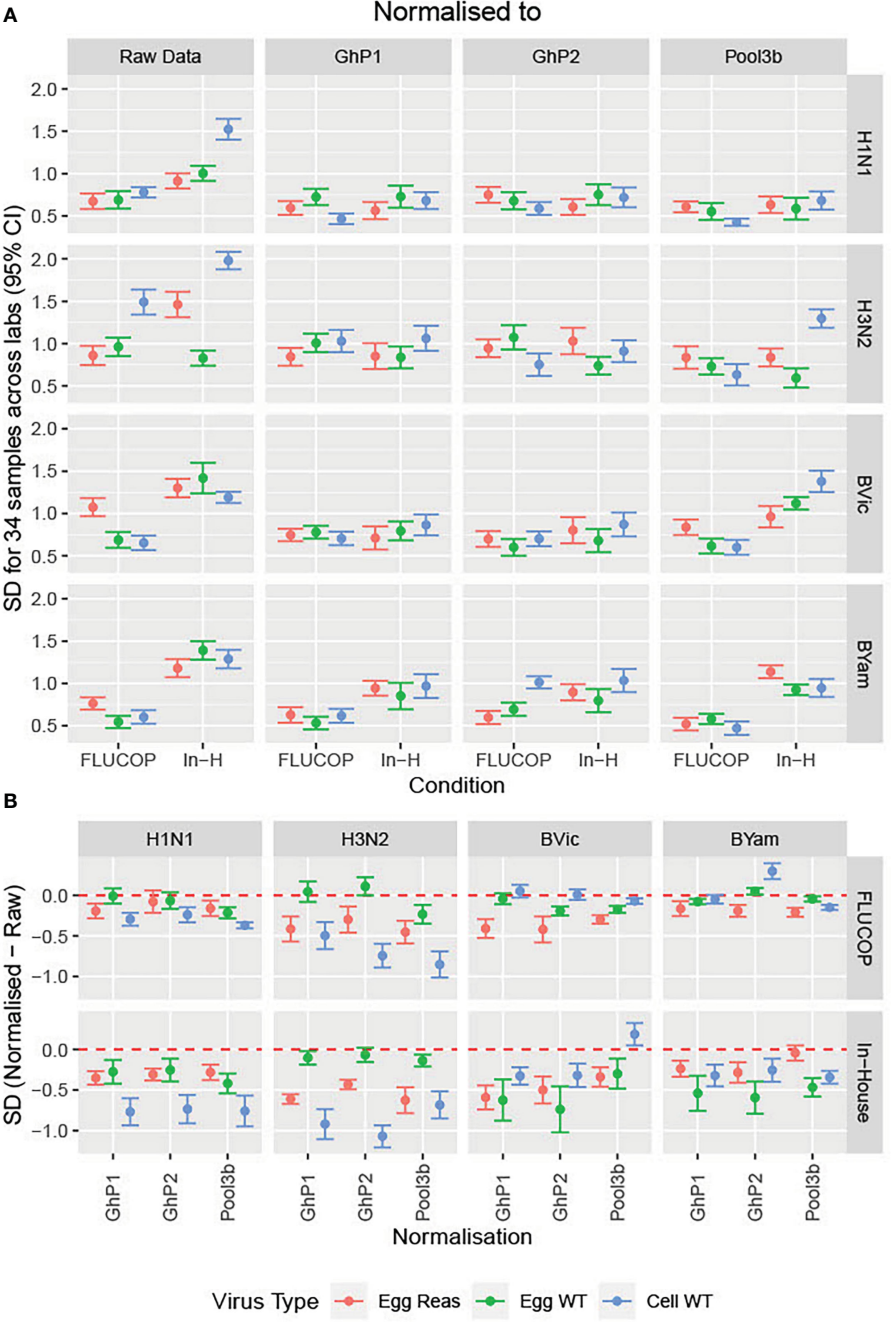
Impact of normalisation on inter-laboratory variation in HAI titres. **[3A]** The SD was calculated across the serum panel tested. SD is shown for each influenza subtype/lineage tested as egg grown reassortant virus (red), egg grown WT virus (green) and cell grown WT virus (Blue). SD is shown before normalisation (Raw Data) and after normalisation with three pools of human sera acting as study standards: GhP1, GhP2 and Pool3b (see **Table 3** for components of each pool). **[3B]** The differences in SD between normalised and raw data are plotted as differences between normalised and raw; values of less than 0 indicate lower SD for normalised titres than absolute titres.

We included three pools of human sera in the testing panels to be used as study standards. 2 pools were made by pooling equal volumes of 4 sera from donors who received the same vaccine as those in the shared serum panel (Ghent pool 1 – GhP1 and Ghent pool 2 – GhP2, QIV). A third pool was included as a standard, containing sera from donors vaccinated with a TIV from 2015-16 containing A/California/07/2009, A/South Australia/55/2014 and B/Phuket/3073/2013 vaccine antigens. Titres were normalised using these three study standards. **Figure 3A** shows the impact of normalisation on SD across the serum panel when different protocols and subtypes/lineages were tested. **Figure 3B** shows the difference in SD between raw and normalised titres for all virus strains, protocols and study standards. Values of less than 0 indicate lower SD for normalised titres relative to raw titres. GhPool 1 and 2 are effective as study standards for all viruses tested by in-house protocols (**Figure 3B**, bottom panel): SD was reduced for 12 out of 12 viruses when normalised with GhPool1 or GhPool2. Pool 3b was taken from the vaccine campaign two years before the matching standard pools. The vaccine used for Pool 3b contained different H1N1 and H3N2 viruses, a matching B/Yamagata virus and had no B/Victoria component. Looking at in-house testing conditions, Pool 3b was not as effective as a study standard as GhPools 1 and 2 for the cell propagated B/Victoria strain tested. It was, however, still effective for all H1N1 and H3N2 antigens, all B Yamagata antigens and B Victoria egg propagated antigens. Values for FLUCOP testing (**Figure 3B**, top panel) are overall less negative than those for in-house testing as the SD for FLUCOP testing is already reduced compared to in-house testing. The exception to this is H3N2 (particularly cell WT antigen) where the SD of consensus testing remains high and is further reduced by normalisation.

**Table 3:** Pools of sera used as study standards: vaccine virus strains.

Pool	Year	H1N1	H3N2	B/Victoria	B/Yamagata
GhPool1	2017-18 (NH)	A/Michigan/45/2015	A/Hong Kong/4801/2014	B/Brisbane/60/2008	B/Phuket/3073/2013
GhPool2	2017-18 (NH)	A/Michigan/45/2015	A/Hong Kong/4801/2014	B/Brisbane/60/2008	B/Phuket/3073/2013
Pool 3b	2015-16 (NH)	A/California/07/2009	A/South Australia/55/2014 (A/Switzerland/9715293/2013-like)	N/A	B/Phuket/3073/2013

NH, Northern hemisphere.

**Figure 4:**
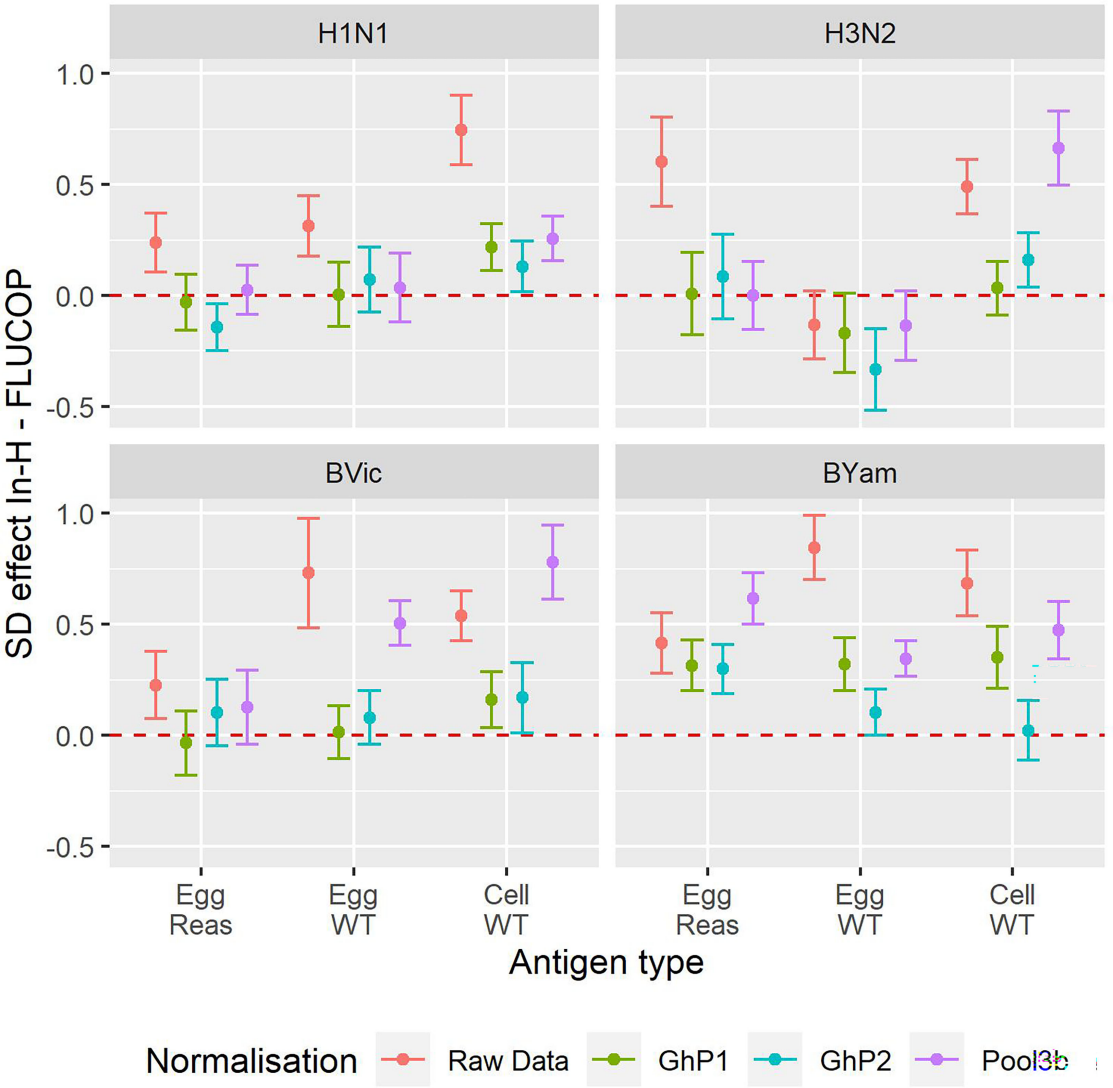
Effect of antigen type, protocol type and normalisation on inter-laboratory variation. Reduction in per sample SD of the log2 titre for In-House versus FLUCOP for all available data from maximally 8 laboratories. Red = Raw Data, Brown = Ghent Pool1, Cyan = Ghent Pool2, Purple = Pool3b. H1N1 Cell WT 7 labs, H3N2 Egg WT 5 labs, BVic Egg WT 7 labs. The red dashed line represents 0 i.e where SD is the same for in-house and FLUCOP testing. Values greater than 0 indicate a higher SD for in-house testing than FLUCOP testing. Overall GMTs for each antigen type, condition and normaliser are shown in **Figure S2**.

We next considered whether normalisation on its own gives the best inter-laboratory agreement, or if FLUCOP testing still provides further reduction in variability. **Figure 4** shows the difference in SD (In-house SD minus FLUCOP SD) – here values higher than 0 show SD is higher for in-house testing than FLUCOP testing. The higher the value, the greater the benefit of using FLUCOP testing. For most antigens before normalisation there is a clear benefit to the use of a consensus protocol and common antigens (see **Figure 4**, data in red). Interestingly after normalisation the benefit of using the FLUCOP protocol/antigen is greatly reduced, however the values remain positive for the B viruses (particularly for B Yamagata), suggesting that even if a commercial seasonal influenza standard was available, assay harmonisation would still reduce inter-lab variation for the B viruses. Values are close to 0 for the A strains, suggesting that there is little cumulative benefit to using both FLUCOP testing and normalisation against a standard.

### 3.2 MN data returned

#### 3.2.1 Selection of FLUCOP protocols for MN testing

Initially the consortium reviewed existing protocols from 11 laboratories within the FLUCOP consortium. Two assay formats were regularly used: an overnight ELISA-based assay and a longer (3-5 day) HA/CPE/ELISA-based assay. Review of these protocols showed that the overnight ELISA format was very consistent across laboratories. It was decided to take forward the WHO protocol (https://www.who.int/influenza/gisrs_laboratory/2010_12_06_serological_diagnosis_of_influenza_by_microneutralization_assay.pdf last accessed 09 January 2023) as the ‘FLUCOP’ protocol for ELISA based MN testing. Assay formats for the longer HA/CPE/ELISA based method showed greater variability. Variable parameters included pre-seeding (or not) of cells, assay length, critical reagents (including serum containing or serum free media, antigen type) and readout methods (HA, CPE and ELISA). A preliminary set of experiments looked at the impact of using a common source (and common growing conditions) of cells and a common source of virus alongside different methods for preparing cells and different assay readouts. From this work a FLUCOP protocol was agreed, presenting a balance between reducing variability and the practicalities of shared resources for testing. A 3-5 day protocol using a laboratory’s own source of cells with an HA/CPE or ELISA based readout (see **Supplementary Materials** for protocol) was chosen for the collaborative study.

#### 3.2.2 Intra-laboratory and inter-laboratory variability for overnight ELISA and 3-5 day format MN assay readouts

A collaborative study was carried out testing 5 viruses (4 High Growth Reassortants (HGRs) representative of the candidate vaccine viruses from 2017-18 and a cell grown H3N2 WT virus representative of non-hemagglutinating viruses, see **Table 1**). Seven laboratories from the FLUCOP consortium tested the WHO ELISA-based MN assay and 3 laboratories tested the FLUCOP 3-5 day assay. Laboratories tested each serum sample in duplicate within a run, and carried out two independent runs.

**Figure 5:**
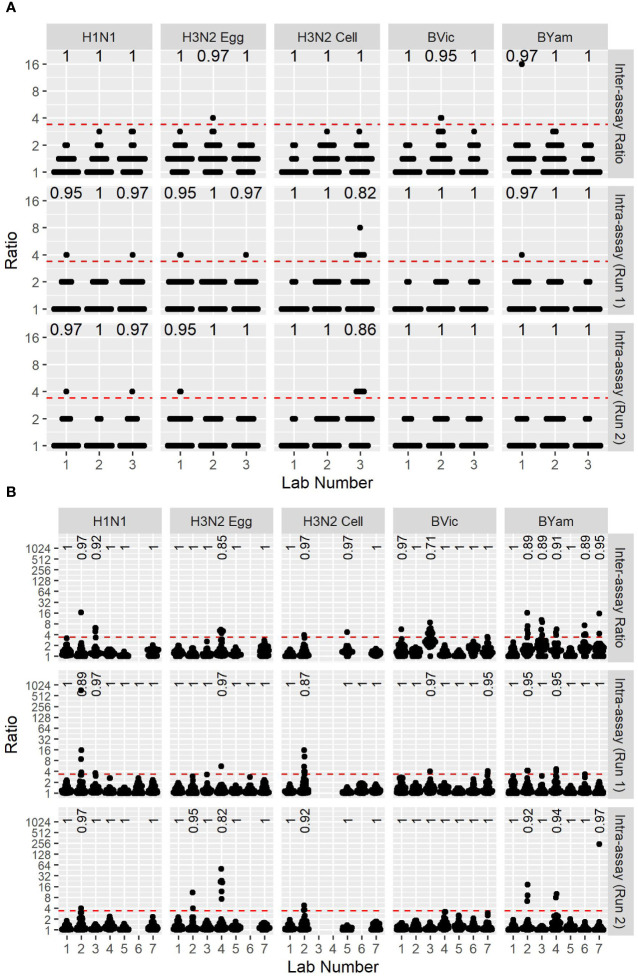
Between-run and within-run variation of two MN assay formats. **[5A]** FLUCOP 3-5 day format MN assay max-min ratios of GMTs between two independent runs (top, Inter-assay ratio) as a measure of inter-assay (or between-run) variability, and max-min ratios of two duplicate samples in run 1 (middle – Intra-assay (Run1)) and run 2 (bottom – Intra-assay (Run2)) as a measure of intra-assay (or within-run) variability. **[5B]** WHO ELISA MN assay max-min ratios of GMTs between two independent runs (top, Inter-assay ratio) as a measure of inter-assay (or between-run) variability, and max-min ratios of two duplicate samples in run 1 (middle) and run 2 (bottom) as a measure of intra-assay (or within-run) variability. Red dashed line represents the ratio cut-off of 3.5. Numbers at the top of each panel indicate the fraction of datapoints < 3.5.

We calculated the maximum-minimum ratios of sample duplicates tested in each run to give a measure of intra-assay (or within-run) variability and the maximum-minimum ratios of sample titres in runs 1 and 2 to give a measure of between-run variability for both the FLUCOP 3-5 day MN assay (see **Figure 5A**) and WHO ELISA format of the MN assay (see **Figure 5B**). For the FLUCOP 3-5 day MN, intra-assay (or within-run) variability was low for all egg viruses tested. The cell propagated H3N2 virus showed greater variability in duplicate samples within a run – if we assume an arbitrary cut-off of max-min ratio >3.5, around 5% of the data would be excluded from titres against cell propagated H3N2 virus across all testing laboratories (compared to ~2.2/2.3% for H1N1 and H3N2 egg propagated viruses, and 0-0.4% for B lineage egg propagated viruses). Between-run variability was broadly consistent across all strains tested (see **Figure 5A**, Inter-assay ratio). For the WHO ELISA-format (see **Figure 5B**), within-run variability was broadly similar for all virus strains tested. The % of duplicate sample titre ratios <3.5 across all testing laboratories in run 1 and run 2 was 98% or above for each influenza A subtype and B lineage tested (with some laboratory specific incidences of higher variability for H3N2 strains). Between-run variability for the WHO ELISA format was consistent for A strains (average % max-min ratios <3.5 97.5-98.0%), however greater variability was observed for the B strains; average % max-min ratios <3.5 dropped to 95.2% for B Victoria and 93.5% for B Yamagata.

**Figure 6:**
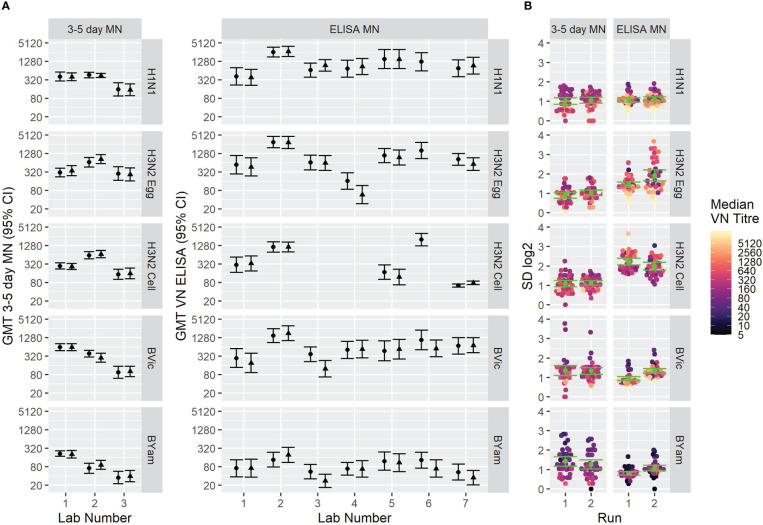
GMTs and inter-laboratory variation for two different MN assay formats. **[6A]** GMTs of 34 samples tested using (left) the FLUCOP 3-5 day MN and (right) the WHO ELISA-based MN. **[6B]** SD of 34 samples tested using (left) the FLUCOP 3-5 day MN and (right) the WHO ELISA MN.

**Figure 6** shows the overall GMTs of the sample panel using the two assay formats (6A) and the SD as a measure of inter-laboratory variation (6B). GMTs do not substantially differ between the two assay formats. SD was lower using the FLUCOP 3-5 day format for H3N2 (egg and cell) antigens and SD was lower for the B strains using the WHO ELISA MN method, albeit with higher inter-assay variability using this method. It should be noted that this is not a direct comparison of laboratories testing both methods simultaneously.

#### 3.2.3 Use of a biological standard to reduce inter-laboratory variability of MN titres

**Figure 7:**
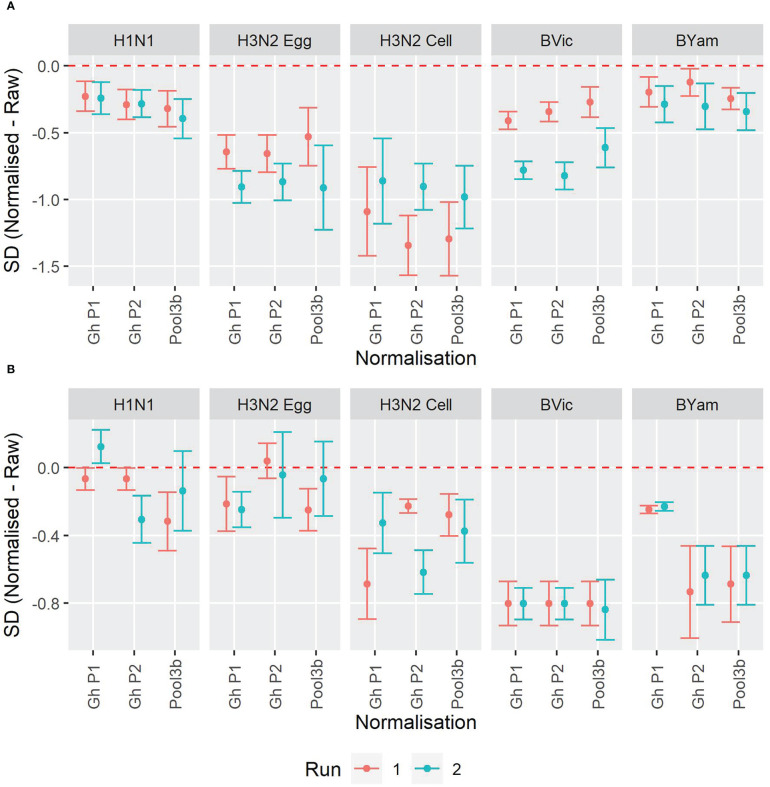
Impact of normalisation on inter-laboratory agreement for WHO ELISA and FLUCOP 3-5 day MN assays. **[7A]** WHO ELISA readout: Change in SD when normalisation is applied. **[7B]** FLUCOP 3-5 day MN: Change in SD when normalisation is applied. Points represent the mean of the per sample pairwise difference of Normalised – Raw with corresponding 95% CI. The red dashed line at 0 indicates no difference.

We included three pools of human sera to be used as study standards. These pools were the same as described for the HAI collaborative study. **Supplementary Figure S3** shows the overall GMTs before and after normalization, and **Figures S4-S5** show the SD for each sample before and after normalisation for the WHO ELISA-based (S4) and FLUCOP 3-5 day (S5) MN assays. **Figure 7** shows the change in SD for each influenza strain tested using both the WHO ELISA assay format (7A) and the FLUCOP 3-5 day assay format (7B). For the WHO ELISA-based assay all strains had significantly reduced SD after normalisation. For the FLUCOP 3-5 day assay, B Victoria, B Yamagata and cell propagated H3N2 had significantly reduced SD after normalisation. For H1N1, SDs were significantly reduced in 4/6 runs, for H3N2, in 3/6 runs. Overall, normalisation significantly reduced inter-laboratory variation in 55/60 runs across the collaborative study.

### 3.3 Correlations between HAI and MN assays

**Figure 8:**
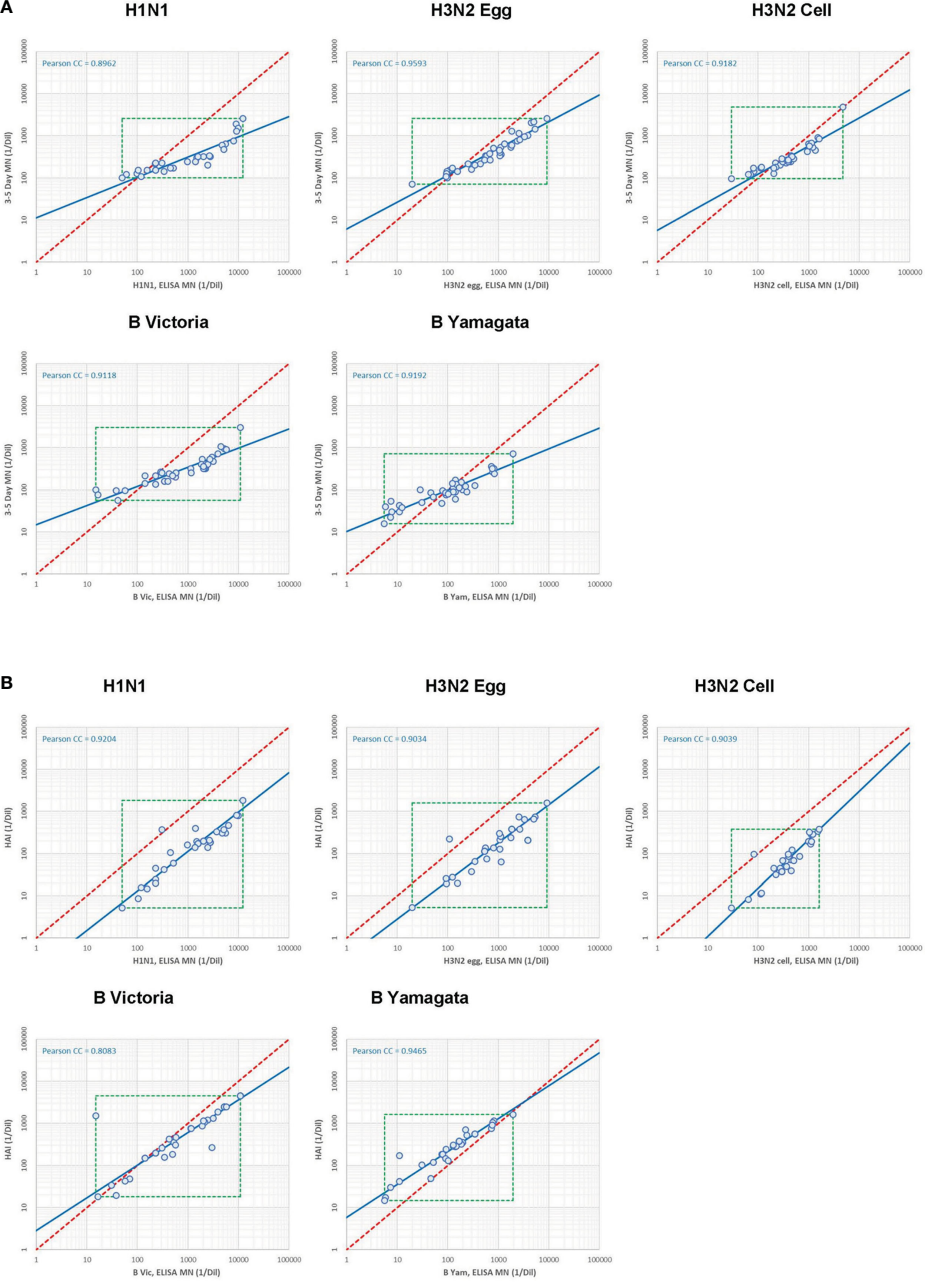
Correlation between serology assays. **[8A]** Correlation between WHO ELISA based (short form, or SF) and FLUCOP 3-5 day (long form or LF) MN assay titres. **[8B]** Correlation between WHO ELISA-based (short form or SF) MN and HAI assay titres.

As labs tested the same serum panel for both the WHO ELISA-based and the FLUCOP 3-5 day-based MN assays, and 28 of these samples were also tested in the HAI study, we were able to look at agreement between MN methods and MN/HAI serology assays. **Supplementary Figure S6** shows the correlation between each method/assay for the 5 viruses tested in the MN study (**Figures S6A-E**). **Figure 8A** shows the relationship between WHO ELISA and FLUCOP 3-5 day MN titres for each of the 5 viruses tested. There was a good correlation for every strain tested (Pearson correlation coefficients of 0.8717-0.9419), however, the titre ratio was not consistent across the dynamic range of the assay and the slope of correlation was substantially lower than 1 (0.41-0.70). At lower titres (~<100) the FLUCOP 3-5 day assay gave higher titres than the WHO ELISA MN, however as titres increase (~>100) the WHO ELISA assay gave higher titres than the FLUCOP 3-5 day MN. Overall, absolute values trended lower in the FLUCOP 3-5 day assay (GMT ratios of 3-5 day FLUCOP assay over WHO ELISA assay for H1N1: 0.28, H3N2 egg: 0.56, H3N2 cell: 0.79, B Vic:0.50, B Yam: 1.05). The dynamic range of the WHO ELISA assay was greater than the FLUCOP 3-5 day assay for each strain tested (ratio of the range WHO ELISA/3-5 day assay (where the range is the ratio of the highest titre/lowest titre in the panel) - H1N1: 9.79, H3N2 egg: 3.84, H3N2 cell: 3.2, B Victoria:13.4, B Yamagta: 4.93). In practical terms the inconsistent titre ratio means that a conversion factor cannot be established, and titres across the different assay formats cannot be directly compared. We assessed if normalisation of the MN titres would improve agreement between the two assay formats. There was no change in correlation after normalisation for each strain (see **supplementary Figure S7**) – the titre ratio remains inconsistent across the dynamic range of the assay.

As long format MN assays include multiple rounds of viral replication, they are thought to possibly capture activity of anti-NA antibodies in addition to the mostly anti-HA antibodies measured in HAI and short ELISA MN methods. We explored this idea by looking at Enzyme-Linked Lectin Assay Neuraminidase Inhibition (ELLA-NI) titres for several samples included in this collaborative study that had been tested in ELLA-NI against the H1N1 virus. Correlations between ELLA-NI, HAI and MN titres for H1N1 can be seen in **Supplementary figure S6A**. **Supplementary figure S8** shows the correlation between ELLA-NI titres and the difference between the FLUCOP 3-5 day MN and the WHO ELISA MN, with an overall negative correlation between ELLA-NI titres and the difference in MN titres. Samples with higher ELLA-NI titres show a greater disparity in MN assay format titres. To build a more accurate model of this complex relationship a larger sample size would be needed, alongside testing within the same laboratory to reduce confounding variables.

We next looked at the correlation between the WHO ELISA MN and HAI assays. 28 samples were included in both HAI and MN collaborative study serum panels. This allowed us to carry out a head-head comparison of HAI and MN titres in multiple laboratories testing all 4 influenza subtype/lineages including a cell grown H3N2 virus. **Figure 8B** shows the correlation between WHO ELISA MN and HAI titres, with a good linear relationship for each strain tested (Pearson correlation coefficients of 0.81-0.95) and the slope of the regression line was close to 1, particularly for the A strains (H1N1: 1.07, H3N2 egg: 1.10, H3N2 cell: 0.87, B Vic: 1.29, B Yam: 1.28). Absolute values trended higher in WHO ELISA MN than HAI for the A strains (GMT ratios were 8.95 (H1N1), 5.46 (H3N2 egg) and 5.41 (H3N2 cell) but are comparable for B strains (GMT ratios were 1.43 (B Vic) and 0.47 (B Yam). The dynamic range of the WHO ELISA MN and HAI assays were similar for the A strains; however the WHO ELISA MN had a larger dynamic range than HAI for the B strains. The good linear relationship, a slope close to 1 and a consistent titre ratio across the dynamic range suggests that HAI and WHO ELISA MN assays are very similar.

## 4 Discussion

Standardised methods for seasonal influenza serology are critical for the continued assessment of seasonal vaccines, the development of novel influenza vaccines and defining correlates of protection against influenza infection. In this study we have continued to drive the standardisation of two commonly used serological methods: the HAI and MN assays.

In a previous FLUCOP study we developed and tested a standardised HAI assay (2) which we have further characterised in this study. We now have an HAI standard operating procedure (SOP) tested with all four seasonal influenza subtypes/lineages, using multiple reassortant and WT strains, including both egg and cell propagated viruses. The panel of 16 viruses tested in this study and the previous study (2) show broadly consistent results. The use of a FLUCOP SOP with a common source of antigen significantly reduces inter-laboratory variation for 11/12 virus strains tested in this study, confirming that a strict level of harmonisation is effective in reducing inter-laboratory variability. We also confirmed the use of a biological standard with in-house testing as an effective tool in reducing inter-laboratory variability, with 12/12 strains having reduced inter-laboratory SD after normalisation with the matching standard GhP1 and demonstrated that a standard is still effective with missmatched strains (here 10/12 strains having reduced inter-laboratory SD after normalisation with a missmatched standard) provided that the standard was generated from individuals vaccinated with a quadrivalent influenza vaccine. This is further evidence, along with the previous FLUCOP HAI collaborative study (2), that such a standard could have a lifespan longer than a single vaccine campaign year.

Our comparison of harmonisation versus normalisation with a standard indicates that harmonisation of B lineage virus preparation and HAI testing would be beneficial, even if a biological standard were available.

In this study we looked at the impact of different antigen sources on HAI titres. Broadly egg grown reassortant and WT viruses had similar GMTs, however the cell propagated viruses gave higher GMTs for the H1N1 strain tested, and lower GMTs for the H3N2 strain tested. The B viruses showed little difference between egg and cell propagated antigens. These strain specific differences are problematic for comparison of antigenically similar/identical egg and cell viruses – these systematic biases in GMT cannot be reduced by harmonisation of protocols, however a biological standard would be ideal for minimising the impact of antigen source on HAI titres.

In the second collaborative study presented here, we sought to harmonise and test two different MN methods. Selecting both the WHO ELISA-based short format and a longer 3-5 day format of the assay allows for the testing of non-agglutinating virus strains, and some flexibility with assay choice depending on time, available equipment and resources. The ability to test non-agglutinating strains is important as recent H3N2 strains have shown reduced binding to both avian and guinea pig RBCs (5–8). Our data indicate that intra-assay (or within-run) performance for both methods was good across the four influenza A subtypes/B lineages tested. Between-run variability was higher for WHO ELISA-based testing of B lineage viruses, particularly B Yamagata.

Whilst an HAI titre of ≥40 has long been described as a correlate of protection, representing 50% protection from infection (22), no such correlate has been attributed to MN titres. The question of whether HAI and MN titres are sufficiently comparable to assign an equivalent correlate for MN titres is complicated. There have been several studies investigating the relationship between HAI and MN titres, the majority focusing on the correlation between methods. Wood et al. (12) and Stephenson et al. (14) showed that whilst there was a positive correlation between HAI and MN titres in most testing labs, it was not possible to assign a ‘conversion factor’ between HAI and MN titres due to strain specific and sample specific differences in the ratio of titres from the two methods. A number of other studies similarly showed positive correlation between the methods, but strain specific differences (23–25), and in one study age-specific differences (26) in conversion factors between the methods. It is generally noted that MN assays are more sensitive than HAI (12, 23, 25). Sicca et al. (23) showed no improvement in agreement between multiple MN methods after normalisation using a sample from the panel tested in the study. To date these studies have either used limited numbers of viruses, limited numbers of testing laboratories, have not used shared panels for testing or have only used limited harmonisation of protocols. Our data agree with these previous studies, suggesting that the shorter ELISA-based MN assay and the longer 3-5 day MN assay correlate well but a conversion factor cannot be established and direct comparison between the two methods is not appropriate. We also observed [like Sicca et al. (23)] that normalisation of MN titres using a biological standard doesn’t improve the agreement between MN methods. These assays may be measuring the contribution of different antibodies; the short form, where the virus does not undergo multi-cycle replication but only infection of cells, will indicate neutralisation from antibodies that block infection of cells (i.e. predominantly anti-HA antibodies). The correlations of ELISA-based MN and HAI assay results are good, and consistent across the dynamic range of the assay, particularly for the A strains tested here. This suggests that a tentative correlate could be assigned to ELISA MN titres (but not 3-5-day CPE MN titres) equivalent to HAI. This would need to be further investigated to assess any strain-specific differences in titre correlation using a wider panel of influenza viruses. ELISA MN and HAI titres may be similar as both assays are likely measuring antibodies that block HA binding. The longer 3-5 day MN method allows for multiple rounds of viral infection. Here the contribution of antibodies against other viral proteins may be measured in addition to anti-HA antibodies. These could include for example the effect of antibodies to M2 protein and internal influenza proteins [such as the NP protein; anti NP antibodies have been shown to help clear infection in mice (27)] which might prevent release of virus and subsequent cycles of infection, anti-NA antibodies or anti-HA antibodies targeting regions other than the globular head of the protein [neutralizing antibodies have been found that bind the membrane proximal stem of HA, such as CR6261 (4) and CR8020 (28)].

## 5 Conclusions

Several studies have demonstrated that strict levels of harmonisation are needed to reduce inter-laboratory variation in HAI titres, although it is possible that the benefit of harmonisation would increase with larger numbers of testing laboratories. Our data support the conclusion that the use of a biological standard is an effective tool for reducing inter-laboratory variability in HAI and MN titres for every strain of influenza tested in this and a previous FLUCOP study (2); 16 influenza strains have been tested in total, across each seasonal Influenza A subtype/B lineage over multiple years, including WT, reassortant, egg and cell cultured strains. Future work should focus on the development of such seasonal influenza standards. While such standards reduce inter-laboratory variability when testing a single method, in this study a standard did not improve agreement between MN serology methods. The non-linearity of the relationship between ELISA MN and 3-5 day MN assay formats remains, and thus a correlate of protection may need to be established for each MN assay method/format independently. Our data do indicate that the ELISA MN and HAI assays are comparable and that a tentative correlate equivalent to HAI could be assigned for the influenza A strains tested in this study. Clinical data supporting this would be of particular importance if such tentative correlates were to be used within regulatory frameworks for novel vaccine development.

## FLUCOP consortium collaborators

Marie-Clotilde Bernard, Barbara Camilloni, Marco Cavaleri, Annalisa Ciabattini, Frederic Clement, Simon De Lusignan, Oliver Dibben, Susanna Maria Roberta Esposito, Felipa Ferreira, Sophie Germain, Sarah Gilbert, Sarah L Jalloh, Stefan Jungbluth, Marion Koopmans, Teresa Lambe, Geert Leroux-Roels, Donata Medaglini, Manuela Mura, Martina Ochs, Albert Osterhaus, Anke Pagnon, Elena Pettini, Leslie Reperant, Sarah Tete, Alexandre Templier, Serge van de Witte, Gwenn Waerlop, Ralf Wagner, Brenda Westerhuis.

## Data availability statement

The raw data supporting the conclusions of this article will be made available by the authors, without undue reservation.

## Ethics statement

The studies involving human participants were reviewed and approved by Universitair Zeikenhuis Gent, Commissie vor medische ethiek (committee for medical ethics) Belgian registration number B670201733136. The patients/participants provided their written informed consent to participate in this study. The animal study was reviewed and approved by NIBSC’s Animal Welfare and Ethics Review Body (AWERB).

## Author contributions

Conceptualisation and study design CC, GP, OE, JW, TO, RC, and DF. Laboratory work: JW, SH, HS-S, BN, CT, SM, FZ, SL, GL, SL, MF, GL, NM, and TO. Data analysis JW, ER, and LZ. Writing and Editing JW, OE, CC, GP, CT, EM, SM, HS-S, MC, RC, ER, LZ, KH and TO. All authors contributed to the article and approved the submitted version.
