# Supplementary material for: Haemagglutination inhibition and virus microneutralisation serology assays: use of harmonised protocols and biological standards in seasonal influenza serology testing and their impact on inter-laboratory variation and assay correlation: A FLUCOP collaborative study
Source: Front Immunol. 2023 Apr 18;14:1155552. doi: 10.3389/fimmu.2023.1155552 (PMC10151801; doi:10.3389/fimmu.2023.1155552)
Supplement: Supplementary file 1 [file DataSheet_1.docx]

**Supplementary figures**

**S1A**

**S1B**

**S1C**

**Supplementary Figure S1. Overall HAI GMTs of all samples tested by lab** **for [S1A]** egg propagated reassortant viruses, **[S1B]** egg propagated WT viruses and **[S1C]** cell propagated WT viruses. See Table 1 for virus details. GMT with 95% CI is plotted for raw data (red), normalised to Ghent Pool 1 (olive), Ghent Pool 2 (green) or Pool3b (purple). The black dotted lines represents the overall GMT across all laboratories.

**S2A**

**S2B**

**S2C**

**Supplementary Figure S2 – Overall HAI GMTS**. GMTs across the serum panel by laboratory for FLUCOP testing (left) and in-house testing (right) for [S2A] egg propagated reassortants [S2B] egg propagated WT and [S2C] cell propagated WT viruses. Shaded area is the range (max to min) of GMTs across the testing labs. Red dashed line is the overall GMT across all testing labs.

**S3A S3B**

**Supplementary Figure S3 – Overall MN GMTs before (Raw Data) and after normalisation (Normalised to GhP1/GhP2/Pool3b)**. Overall GMTs across the serum panel by laboratory for **[S3A]** FLUCOP 3-5 day MN assay and **[S3B]** WHO ELISA MN assay. Run 1 is shown as a circle, run 2 is shown as a triangle. Range of GMT across labs is shaded in grey.

**Supplementary Figure S4. The impact of normalisation on WHO ELISA MN assay between laboratory agreement.** The SD of 34 samples per run and assay strain are shown across all 6 testing labs, the colour of the points indicates the median well number of the end-point titre. Green points indicate mean SD with 95% confidence intervals.

**Supplementary Figure S5. The impact of normalisation on FLUCOP 3-5 day MN assay between laboratory agreement.** The SDs of 34 samples per run and strain are shown across all 3 testing labs, the colour of the points indicates the median well number of the end-point titre. Green points indicate mean SD with 95% confidence intervals.

**S6[A]** H1N1


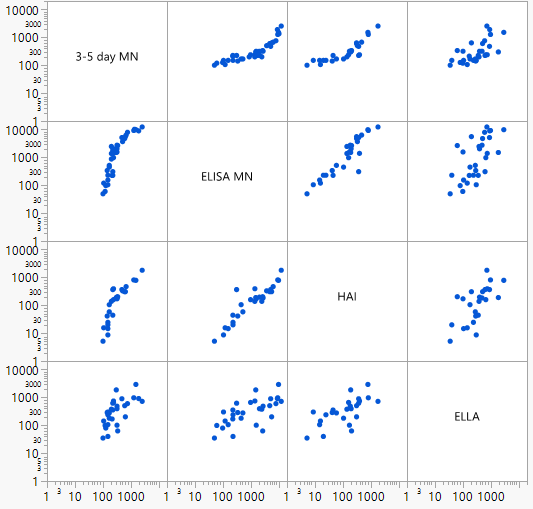


|  | 3-5 dayMN | ELISA MN | ELLA | HAI |
| --- | --- | --- | --- | --- |
| 3-5 dayMN | 1.0000 | 0.9285 | 0.5009 | 0.9704 |
| ELISA MN | 0.9285 | 1.0000 | 0.5532 | 0.8862 |
| ELLA | 0.5009 | 0.5532 | 1.0000 | 0.4602 |
| HAI | 0.9704 | 0.8862 | 0.4602 | 1.0000 |

**S6 [B]** H3N2 Cell


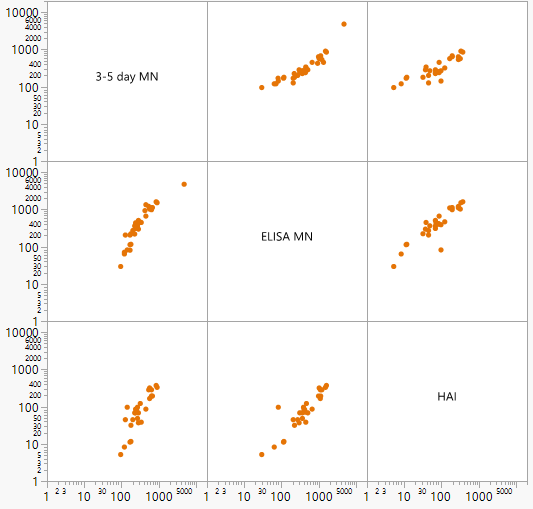


|  | 3-5 day MN | ELISA MN | HAI |
| --- | --- | --- | --- |
| 3-5 day MN | 1.0000 | 0.9505 | 0.8677 |
| ELISA MN | 0.9505 | 1.0000 | 0.9610 |
| HAI | 0. 8677 | 0.9610 | 1.0000 |

**S6[C]** H3N2 Egg


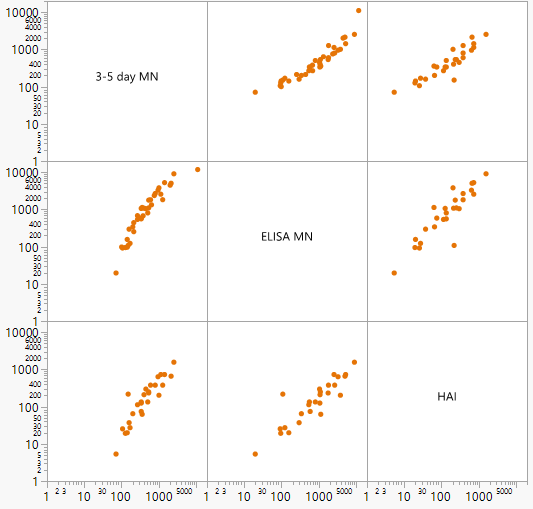


|  | 3-5 day MN | ELISA MN | HAI |
| --- | --- | --- | --- |
| 3-5 day MN | 1.0000 | 0.8575 | 0.9389 |
| ELISA MN | 0.8575 | 1.0000 | 0.9388 |
| HAI | 0.9389 | 0.9388 | 1.0000 |

**S6[D]** B/Victoria


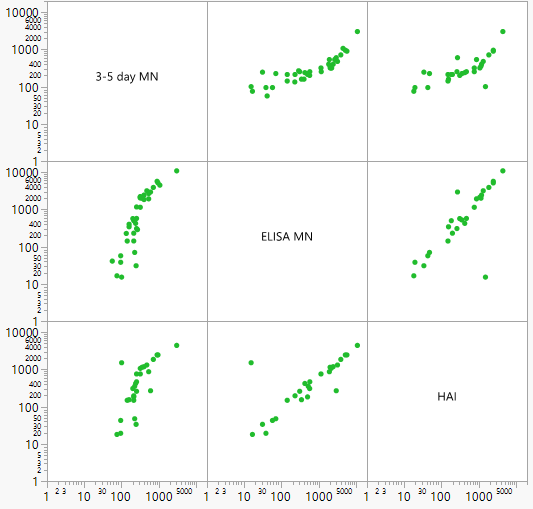


|  | 3-5 day MN | ELISA MN | HAI |
| --- | --- | --- | --- |
| 3-5 day MN | 1.0000 | 0.9424 | 0.8673 |
| ELISA MN | 0.9424 | 1.0000 | 0.9260 |
| HAI | 0.8673 | 0.9260 | 1.0000 |

**S6[E]** B/Yamagata


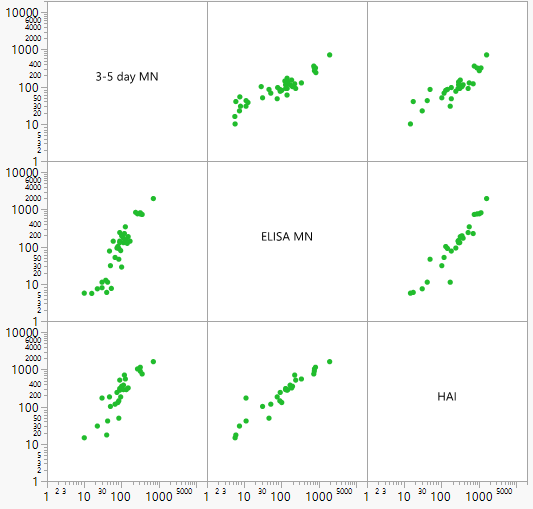


|  | 3-5 day MN | ELISA MN | HAI |
| --- | --- | --- | --- |
| 3-5 day MN | 1.0000 | 0.9693 | 0.8803 |
| ELISA MN | 0.9693 | 1.0000 | 0.9305 |
| HAI | 0.8803 | 0.9305 | 1.0000 |

**Supplementary Figure S6. Correlations and Pearson Correlation Coefficients between 3-5 day MN, ELISA based MN and HAI assay titres**. Data plotted are the GMTs of all valid results by all participating laboratories for **[S6A]** H1N1, **[S6B]** H3N2 cell propagated, **[S6C]** H3N2 egg propagated, **[S6D]** B Victoria and **[S6E]** B Yamagata strains. In each panel the titres are plotted for every combination and Pearson Correlation Coefficients are shown in the table below.

**Figure S7: Linear Relationship between ELISA MN vs. 3-5 day MN before and after normalisation with a study standard.**

|  | | H1N1 | H3N2 (egg) | H3N2 (Cell) | B Vic | B Yam |
| --- | --- | --- | --- | --- | --- | --- |
| **[A]**  **No Normalisation** | **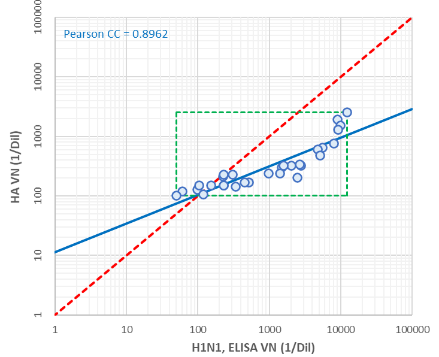** | | **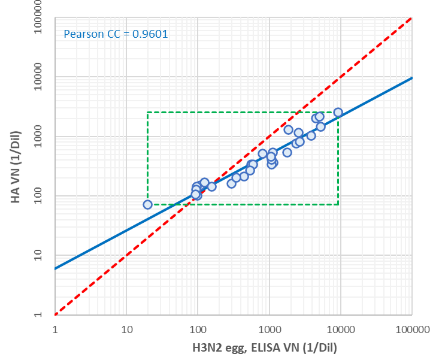** | **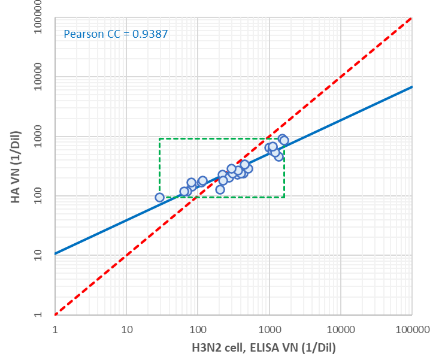** | **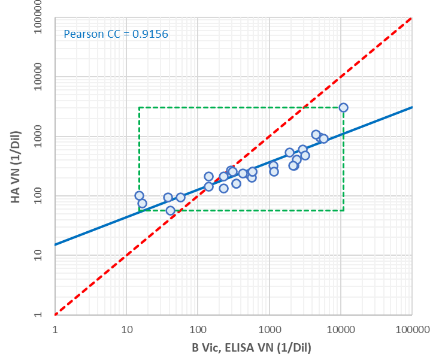** | **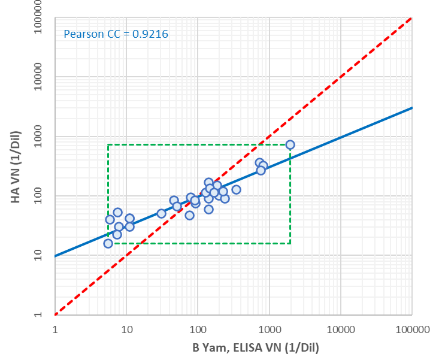** |
| **[B]**  **Normalised against Gh P1** | **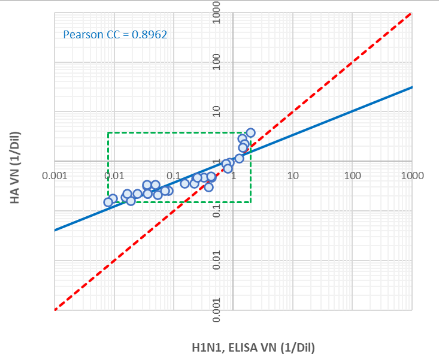** | | **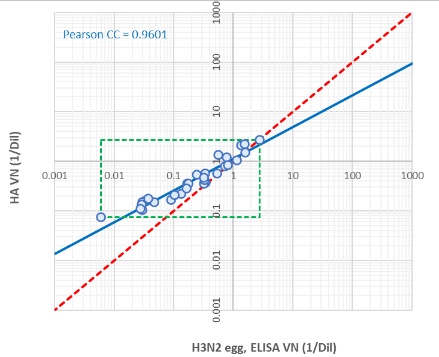** | **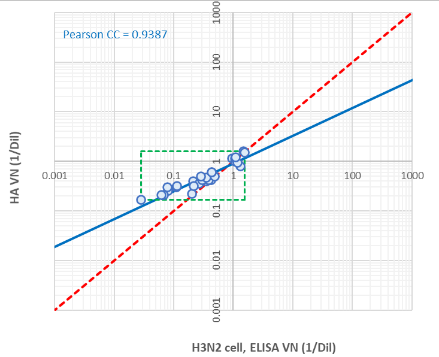** | **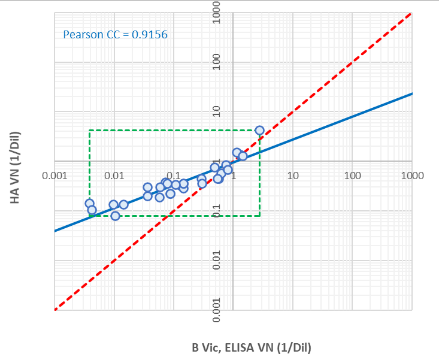** | **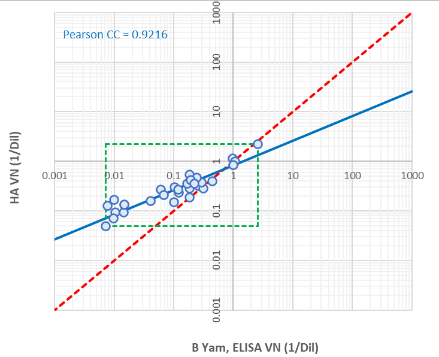** |
| **[C]**  **Normalisaed  against Gh P2** | **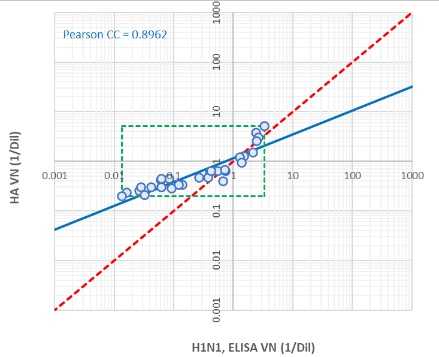** | | **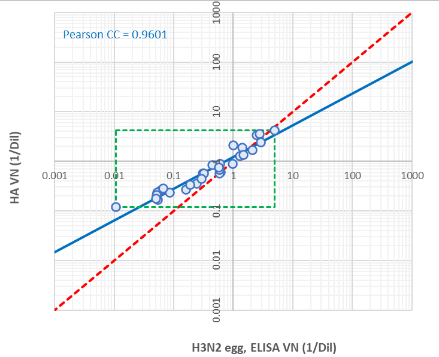** | **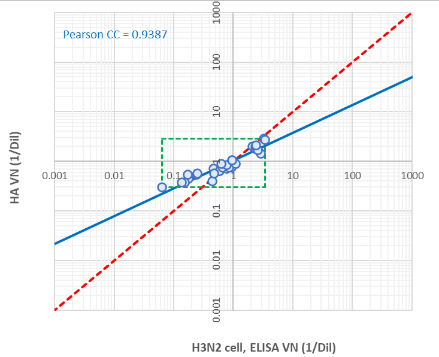** | **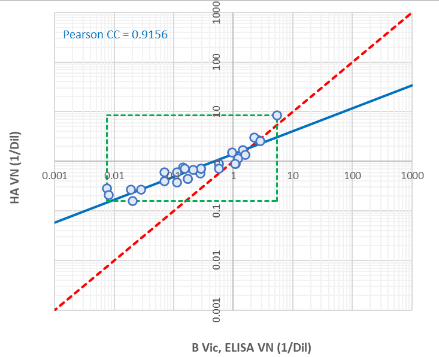** | **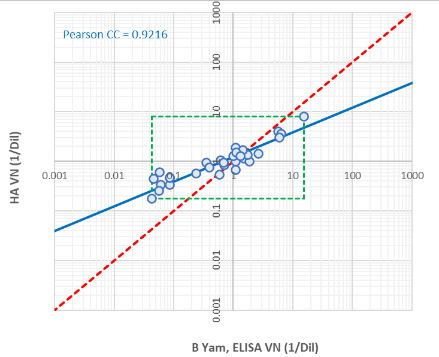** |
| **[D]**  **Normalised against Pool3b** | **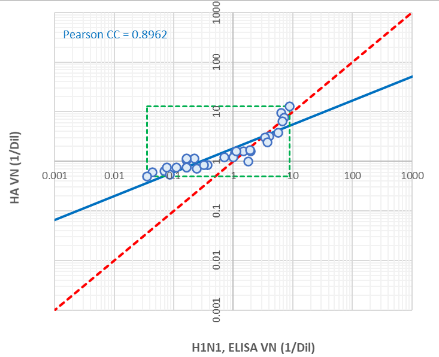** | | **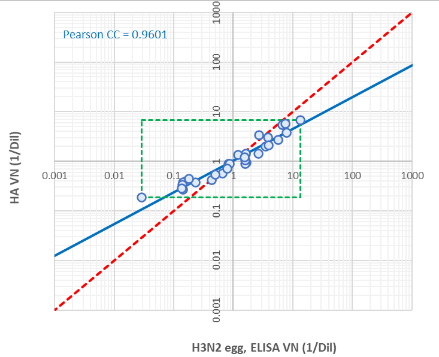** | **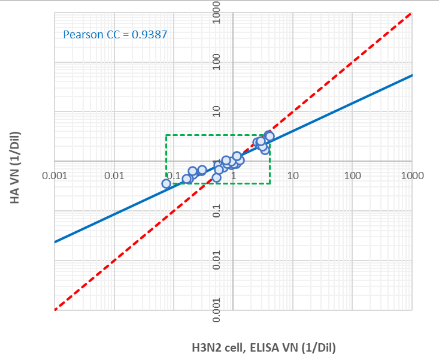** | **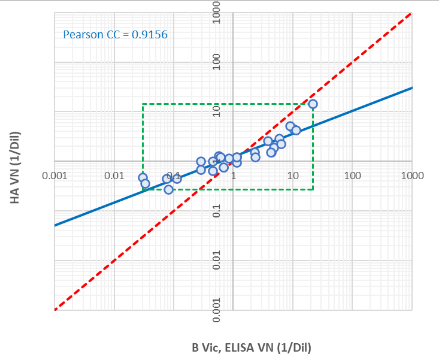** | **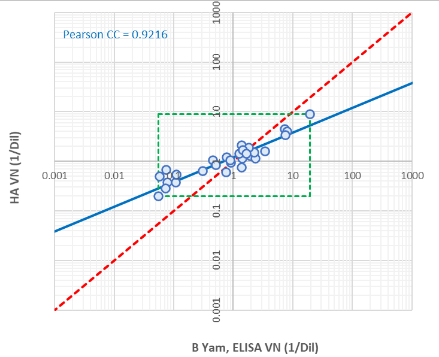** |

**Supplementary Figure S7. Correlation between ELISA MN and 3-5 day MN titres before and after normalisation with a study standards.** MN titres as measured using the ELISA MN are plotted against MN titres measured using the 3-5 day MN for each virus tested. The correlation is shown [A] before normalisation and after normalisation with three pools of post vaccination human sera (see table 3 for details) [B] Ghent pool 1 (GhP1) [C] Ghent pool 2 (GhP2) and [D] Pool3b. Pearson correlation coefficients are shown in each graph.

**S8**


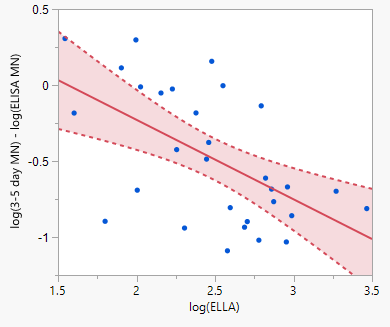


**Supplementary figure S8. Correlation of ELLA-NI titres to difference between 3-5 day MN and ELISA MN titres**. Correlation of log ELLA-NI titres with the difference in ELISA MN and 3-5 day MN titres is plotted. The negative correlation is statistically significant (Analysis of variance P=0.0011*), indicating that as ELLA-NI titres increase, the disparity between 3-5day MN and ELISA MN formats increases.
