# Supplementary material for: Haemagglutination inhibition and virus microneutralisation serology assays: use of harmonised protocols and biological standards in seasonal influenza serology testing and their impact on inter-laboratory variation and assay correlation: A FLUCOP collaborative study
Source: Front Immunol. 2023 Apr 18;14:1155552. doi: 10.3389/fimmu.2023.1155552 (PMC10151801; doi:10.3389/fimmu.2023.1155552)
Supplement: Supplementary file 2 [file DataSheet_2.docx]

Long-format (3-5 day) Microneutralisation (MN) Assay – FLUCOP protocol

**Assay description**

This protocol describes the testing of human sera samples to quantify neutralising anti-influenza antibodies using MDCK cells using a haemagglutination (HA)-based, cytopathic effect (CPE)-based) or ELISA-based readout.

In brief, viruses for testing are first titrated to determine the dilution required for a fixed infectious dose of 100TCID50/50µl. Sera of interest are serially diluted 2-fold across a plate and mixed with the fixed infectious dose of virus. MDCK cells are added to serum-virus mixtures and incubated for 5 days. The presence or absence of virus is measured using one of the following readouts: HA of avian or mammalian red blood cells (RBCs); the presence of CPE; ELISA to detect presence of virus. Wells containing neutralising antibodies will have no viral infection and replication, and wells with without neutralising antibodies will have viral infection and replication. The microneutralisation (MN) titre of the serum sample is determined as the reciprocal of the highest dilution at which virus has been neutralised and no viral infection and replication has occurred i.e., the reciprocal of the highest serum dilution where a well has been scored as negative.

**Handling and preparation of serum and virus**

For long term storage, serum samples should remain frozen at -20°C. Samples should be thawed to room temperature and heat inactivated for 30 mins at 56°C. Serum samples can be stored at 2-8°C for up to 28 days. 10µl of serum are required for each virus to be tested.

Virus should be aliquoted and stored at -70°C to avoid repeat freeze-thaw cycles. A new aliquot of virus should be thawed for each microneutralisation assay on the day of use.

Live virus should be handled at BSL2 level in accordance with in-house SOPs.

| Protective equipment and clothing | Working equipment and materials | Reagents and disinfectants |
| --- | --- | --- |
| Microbiological safety cabinet (MSC) | 37°C CO_2_ incubator | 70% ethanol (disinfectant) |
| Disposable gloves | Pipetboy (or similar) and Serological pipettes | MDCK cells and growth media (Ultra-MDCK Lonza #12-749Q, 2mM L-glutamine) |
| Lab coat | Refrigerated centrifuge | Test Diluent: UltraMDCK (Lonza #12-749Q, antibiotics (P/S) and antimycotics (amphotericin), 2mM L-glutamine. Add 2µg/ml TPCK-treated Trypsin on the day of use. |
| Spray bottle for ethanol disinfectant | Micropipettes, Multichannel pipettes, sterile filtered tips and sterile troughs | Trypsin-EDTA (Lonza, BE17-161E or equivalent) |
| Autoclave bags, autoclave tape, tip disposal container and autoclave boxes | 96 well flat-bottomed cell culture plates | Turkey Red Blood Cells (RBCs) or Guinea Pig RBCs |
|  | 96 well V-bottomed plates (Greiner 651101 or equivalent) | DPBS (without calcium or magnesium Lonza #17-512 or equivalent) |
|  | 56°C water bath/incubator | Fixative: Cold 80% Acetone in PBS (prepared the day before and stored at -20°C or prepared fresh and stored at -40-80°C for at least 10 mins) |
|  | Hemacytometer or automated cell counter, slides and trypan blue | Wash/Blocking buffer: 1% BSA, 0.1% Tween 20 in PBS |
|  | 50ml Falcon tubes (or equivalent) or sterile glass wear (for RBC washing, RBC suspensions and virus working dilutions) | Antibodies: 1°antibody- For A strains - CDC, catalog# VS2208, the recommended reagent from the WHO protocol, or its equivalent Millipore, catalog# MAB8251, clone A1/A3 Blend (MAB8257 + MAB8258). For B strains, mab clone B017, that can be purchase from either Abcam, catalog# ab20711, Santa Cruz Biotechnology, catalog# sc-57885, or BioRad (Serotec), catalog# MCA403  2°antibody - Goat anti-mouse IgG conjugated to horseradish peroxidase (HRP) (Seracare KPL cat # 474-1802 or equivalent). Dilute 1:2000 in blocking buffer or to optimal concentration. |
|  | T75 tissue culture flasks (or similar) | Substrate:  TMB (3, 3’, 5, 5’-Tetramethylbenzidine), Single Component-Soluble Substrate for HRP, Europa Bioproducts Ltd, cat no. MO701A. OR  SureBlue™ TMB 1-Component Microwell Peroxidase Substrate, Seracare KPL, material# 5120-0077 |
|  |  | Stop solution: Hydrochloric acid 0.5 mol (0.5M HCL), BDH cat no. 190696B or Fisher Scientific cat no. J/4330 |

# Prior to neutralisation testing – all steps carried out in an MSC

1. **Titration of viruses**

In a 96 well flat-bottomed cell culture plate set up virus titrations in quadruplicate:

- Add 100ul of test diluent to Columns 2 to 12 of a 96 well plate
- Add 200ul virus to Column 1
- Titrate 100ul across from Column 1-12 (Note: Change tips between each column) and discard the final 100ul
- Place at 37°C for 1 hour (mimics neutralisation step of assay)
- Add 100µl of MDCK cells at 1.5 X 10^5^ cells/ml (see step 4-5. Preparation and addition of MDCK cells)

On day 3-5 carry out preferred readout method (see step 6/7/8 for alternatives)

Calculate the TCID_50_/ml and dilutions required to make a working stock of 100TCID_50_/50µl See Lei et al (Lei et al., 2021) for a summary of TCID_50_ calculation methods.

# Virus Microneutralisation assay – all steps carried out in an MSC

1. **Split MDCK cells (day -1)**

Split a confluent monolayer of MDCK cells one day before use at 1:2 or 1:3 (on the day of seeding 96 well plates (day 0), MDCK monolayer should be 70-95% confluent)

1. **Serum – virus neutralisation step (day 0)**

Prepare a working dilution of the testing virus at 100TCID_50_/50µl in test diluent (5ml is required per plate)

It is recommended that each serum sample is tested in duplicate on separate plates. In a 96 well flat-bottomed assay plate carryout a 2-fold titration of sera to be tested:

- Add 50µl of test diluent to all wells of the plate
- Add an additional 40µl of test diluent to column 1
- Add 10µl of sera to column 1 (final dilution of 1:10) in duplicate (preferably on separate plates if possible)
- Titrate 50ul across the plate from Column 1-11 and discard the final 50µl
- Add 50µl of the working dilution virus (100TCID_50_/50µl) to each well of Columns 1-11 and Column 12 A-D (virus control).
- Add 50ul of test diluent to Column 12 E-H (cell control)

A back titration (BT) of the virus should be carried out in **each** assay run:

- On a separate plate add 50µl of test diluent to Columns 1 to 12 of a 96 well plate
- Add 50µl of the working dilution of virus (100TCID_50_/50µl) to Column 1
- Titrate 50µl across from column 1-12 (Note: Change tips between each column) and discard the final 50µl. The wells will contain: A1: 50 TCID50; B1: 25 TCID50; C1: 12.5 TCID50; D1: 6.3 TCID50; E1: 3.2 TCID50; F1: 1.6 TCID50; G1: 0.8 TCID50; H1: 0.4 TCID50.

***Place both assay plates and virus back titration plate in a 37°C CO_2_ incubator for 1 hour***

|  |  | **1:10** | **1:20** | **1:40** | **1:80** | **1:160** | **1:320** | **1:640** | **1:**  **1280** | **1:**  **2560** | **1:**  **5120** | **1:**  **10240** |  |
| --- | --- | --- | --- | --- | --- | --- | --- | --- | --- | --- | --- | --- | --- |
|  |  | 1 | 2 | 3 | 4 | 5 | 6 | 7 | 8 | 9 | 10 | 11 | 12 |
| **S1** | A |  |  |  |  |  |  |  |  |  |  |  | VC |
| **S2** | B |  |  |  |  |  |  |  |  |  |  |  | VC |
| **S3** | C |  |  |  |  |  |  |  |  |  |  |  | VC |
| **S4** | D |  |  |  |  |  |  |  |  |  |  |  | VC |
| **S5** | E |  |  |  |  |  |  |  |  |  |  |  | CC |
| **S6** | F |  |  |  |  |  |  |  |  |  |  |  | CC |
| **S7** | G |  |  |  |  |  |  |  |  |  |  |  | CC |
| **S8** | H |  |  |  |  |  |  |  |  |  |  |  | CC |

Example of an assay plate: 8 sera are tested in singleton. Column 12 contains virus and cell controls.

1. **Preparation of MDCK cells (day 0)**

Whilst virus or virus-serum is incubating at 37°C for 1 hour, prepare the MDCK cells:

- Wash 70-95% confluent monolayers of MDCK cells 2X with DPBS
- Wash cells with Trypsin-EDTA
- Add 2ml of Trypsin-EDTA for a T75 flask (or equivalent)
- Place at 37°C until the monolayer detaches
- Wash cells 2X with test diluent (spin cells at 485g for 5 mins to pellet)
- Resuspend cells in 10ml/T75 flask (or equivalent) in test diluent and count using a haemocytometer or automated cell counter
- Adjust cell number to 1.5 X 10^5^ cells/ml in test diluent (100µl of this suspension will be added/well in the assay)

1. **Addition of MDCK cells to serum-virus (day 0)**

- Remove assay plates from 37°C incubator
- Add 100µl of MDCK cells (at 1.5 X 10^5^ cells/ml) to each well of the assay plate
- Place plates in a CO_2_ incubator at 37°C for 3-5 days

There are three alternative readout methods (6) an HA-based readout, (7) a CPE-based readout and (8) an ELISA-based readout.

1. **HA Readout of assay (day 3-5)**

Prepare a 0.5% Turkey Red Blood Cell (TRBC) suspension:

- Wash TRBCs 3X using DPBS
  - pellet cells at 500g for 10 mins, 4°C
  - resuspend in DPBS
  - pellet cells at 500g for 5 mins, 4°C
  - repeat above 2 steps
  - pellet cells at 500g for 10 mins, 4°C
  - prepare 0.5% TRBC suspension
- Transfer 50µl/well of supernatant from the assay plate to a 96-well V-bottomed plate
- Add 50µl 0.5% TRBC suspicion into each well
- Tap the plate gently and incubate at RT for 30 minutes

*Scoring*

- Wells with agglutinated or partially agglutinated RBCs are scored as positive. Wells with non-agglutinated RBCs are scored as negative. The MN titre is the reciprocal of the highest dilution scored as negative (i.e. complete neutralisation of the infectious dose of virus and non-agglutinated cells).
- Patterns of completely, partially and non-agglutinated TRBCs are shown below: The virus control and cell control Colum 12 will facilitate the calling of agglutinated/non-agglutinated RBCs.


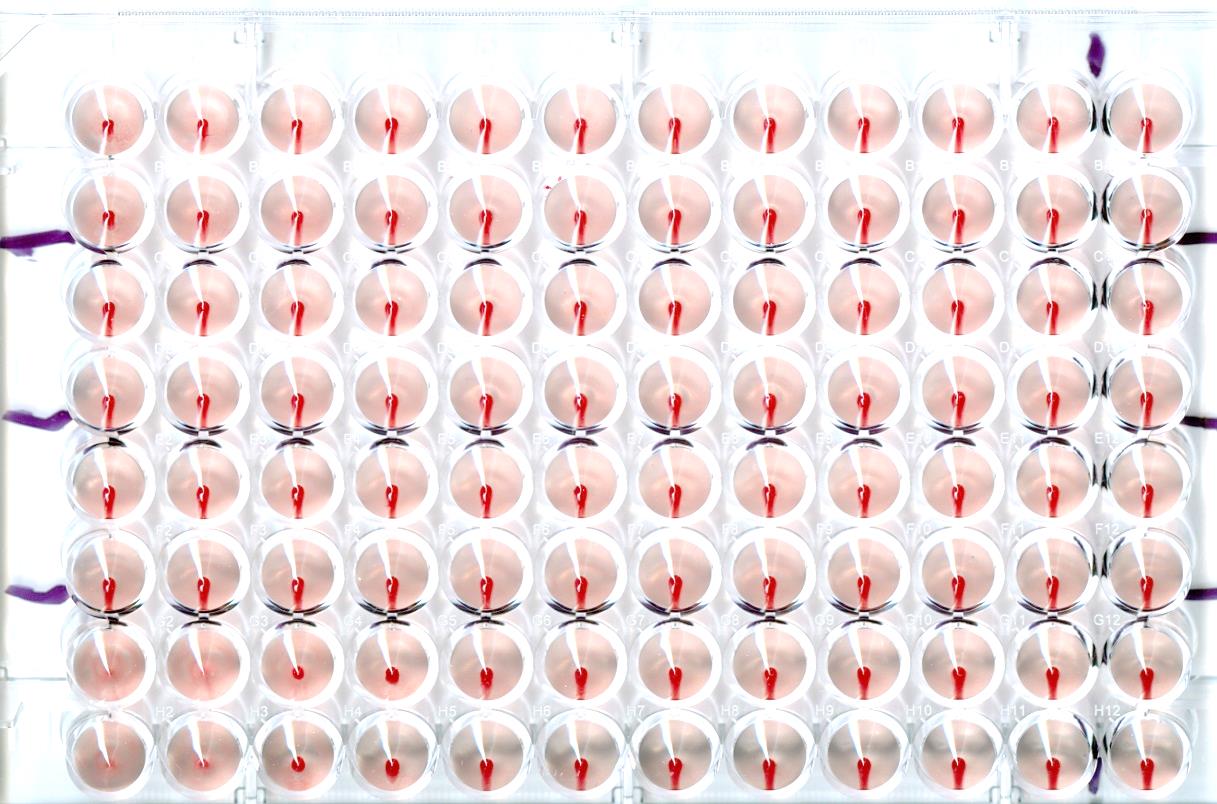


Positive Negative

- Recent H3N2 strains have shown reduced avidity to avian RBCs – Guinea Pig Red Blood Cells (GPRBCs) can be used (0.5% suspension in DPBS, 1% BSA, washing method as described for TRBCs). GPRBCs can be used as described for TRBCs with the exception of a 1h incubation at RT as the cells take longer to fall out of suspension. Patterns of agglutinated and non-agglutinated GPRBCs are different to TRBCs – non agglutinated cells often do not run, but form a button, or a cross shape after tilting. A GPRBC only control should be read at the same time as an assay plate to facilitate calling of agglutinated, partially agglutinated and non-agglutinated GPRBCs.

Non-Agglutinated GPRBCs

**
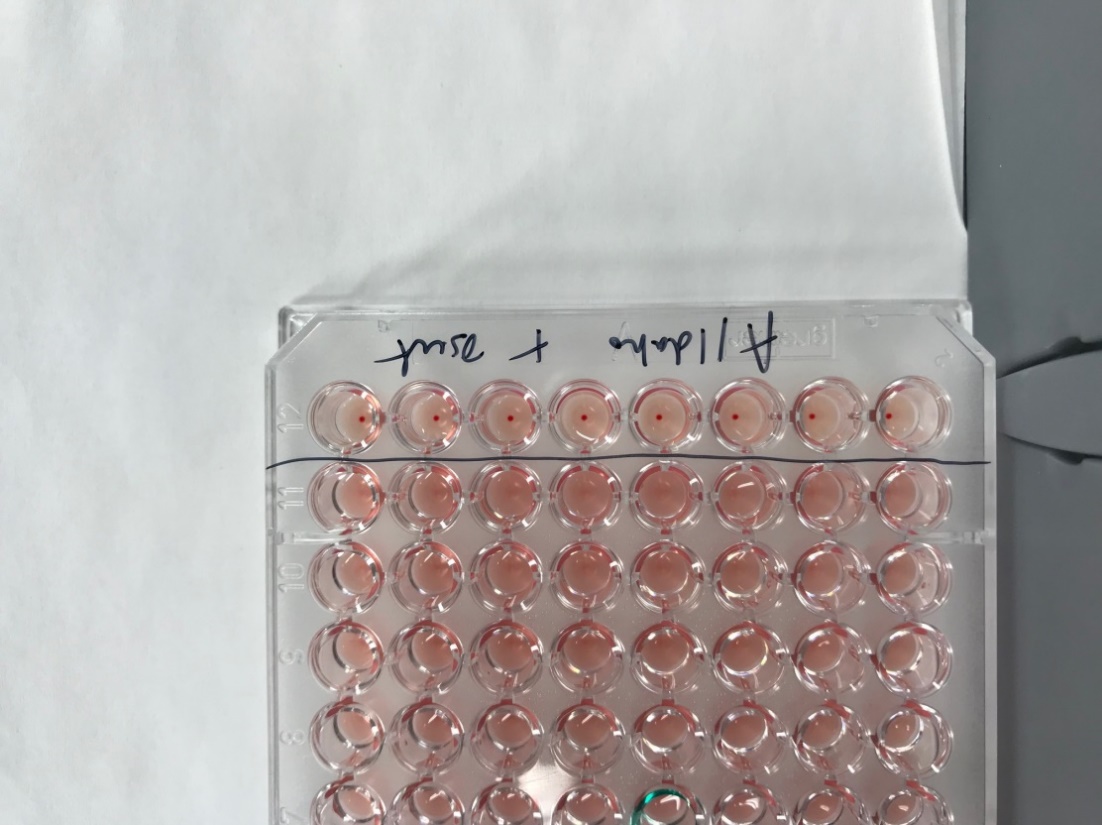
**

**
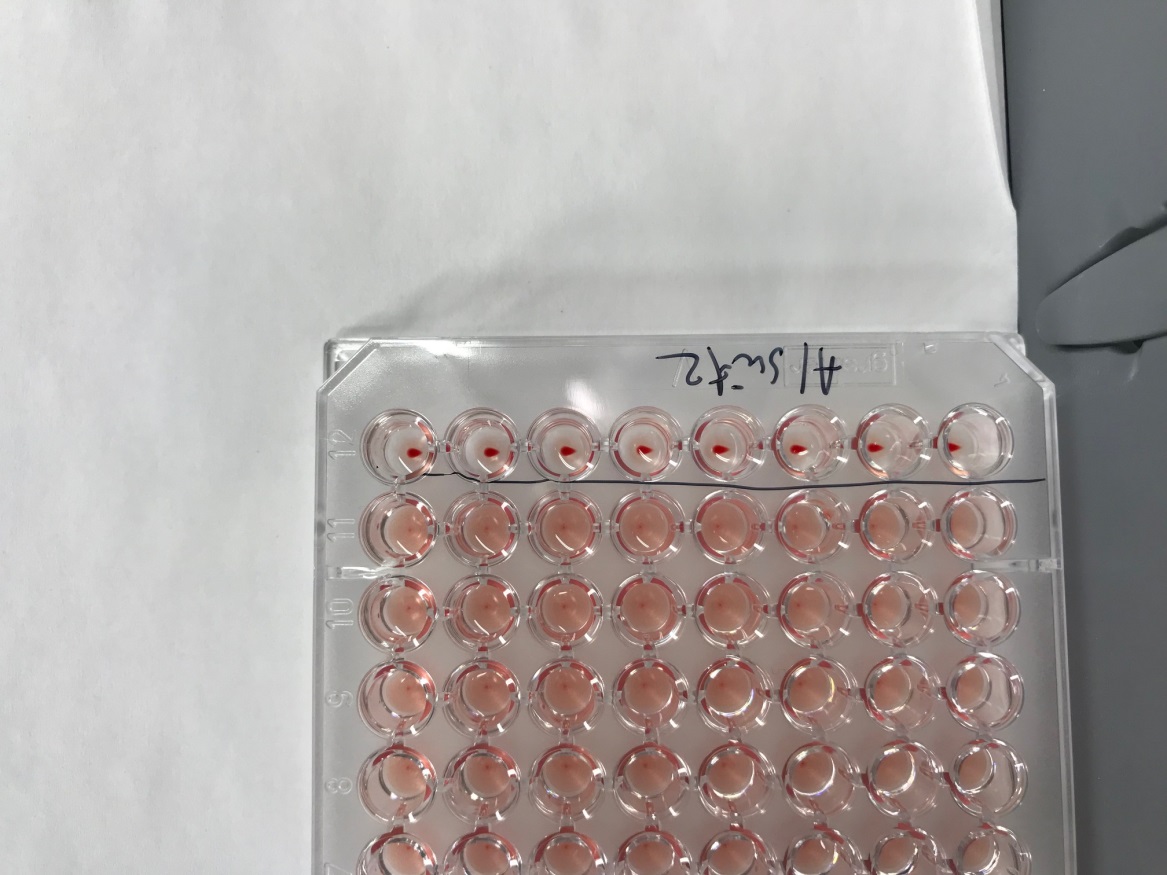
**

Agglutinated GPRBCs

1. **CPE readout of the assay (day 3-5)**

At the end of the incubation period, each well of the 96-well microtiter plate is checked under an optical microscope to assess the presence of local lesions (‘CPE’) in the cell monolayer.

*Definition of CPE*

• Complete destruction of the cell monolayer in the well, or

• Presence of hole(s) in the cell monolayer, surrounded by destroyed cells

Note: An incomplete or not overgrown cell monolayer with hole(s) surrounded by intact cells is not regarded as CPE.

*Definition of Cell control*

• The cell monolayer is intact, meaning that the cells have not been infected.

*Scoring*

• The wells which show CPE are designated “positive” (infected), while the wells which don’t show CPE are designated “negative” (protected). The MN titre is the reciprocal of the highest dilution scored as negative.

1. **ELISA readout (day 3-5)**

Fixation of the Plate(s)

• At the end of the incubation period, remove medium from plate.

• Wash each well once with 200 µl of PBS.

• Remove PBS (do not allow wells to dry out) and add 100 µl ice-cold fixative to each well.

• Cover with lid and incubate at room temperature (RT) for 10 min.

• Remove fixative and let plate air-dry.

• Spray paper towel with 70 % EtOH and wipe exterior of plates before removing from class II biological safety cabinet.

1° Antibody

• Dilute 1°-antibody (anti-Influenza NP mouse monoclonal) in blocking buffer to pre-defined dilution.

• Prepare 10 ml per plate.

• Wash plate(s) 3 x with wash buffer, 150-300 µl per well.

• Add diluted 1°-antibody to each well (100 µl / well).

• Cover plate(s) and incubate for 1 hr at room temperature (RT).

2° Antibody

• Dilute 2°-antibody (goat anti-mouse IgG; HRP conjugated) in blocking buffer to pre-defined dilution.

• Prepare 10 ml per plate.

• Wash plate(s) 3x with wash buffer, 150-300 µl per well.

• Add diluted 2°-antibody to each well (100μl/well).

• Cover plate(s) and incubate for 1 hr at RT.

Substrate

• Warm up TMB to room temperature

• Wash plate(s) 3x with wash buffer, 150-300 µl per well.

• Add TMB to each well (100 µl/well).

• Incubate at RT for 20 min.

• Add 100 µl stop solution to all wells.

• Read absorbance (optical density [OD]) of wells at 450 and 620 (or 630) nm. If not performed automatically by the reader, final OD value is calculated by subtracting 620(630) nm values from the 450 nm values.

• The reading should be performed as soon as possible after addition of the stop solution

Scoring

• Calculations are determined for each plate individually.

• Determine the virus neutralization antibody 50% titre of each serum using the following equation:

x = [(average OD of VC wells) – (average OD of CC wells)]/2

• Where x is the OD value at which 50% of the MDCK cells were infected.

• The wells where OD values are below or equal to x are designated “negative” (protected), while all other wells are designated “positive” (infected).

• The reciprocal of the highest serum dilution scored negative is the 50% neutralization antibody titre

• Each plate is considered acceptable when:

- The cell control has an OD < 0.2.
- The virus control has an OD > 0.8.

• Each run is considered acceptable when:

- The virus test dose (100x TCID) is confirmed by virus back titration.
- The OD of the negative serum control is similar to that observed for the virus control

1. **TCID50 calculation**

(Lei et al., 2021) gives a description of TCID50 calculations typically used. The Improved Kärber Method is recommended.

1. **Acceptance criteria**

- A run is considered acceptable when the virus test dose (100 TCID50) is confirmed by back titration. The acceptable range is 30 to 300 TCID50
- A sample titre is considered acceptable when duplicate titres are within 4-fold. If titres differ by 4-fold or more, the sample should be re-tested (it is recommended to re-test no more than 2 times).

**References**

Lei, C., Yang, J., Hu, J., and Sun, X. (2021). On the Calculation of TCID(50) for Quantitation of Virus Infectivity. *Virol Sin* 36**,** 141-144.

**Glossary**

MN - Microneutralisation

CPE – cytopathic effect

HA - haemagglutination

SOP – standard operating procedure

MSC – microbiological safety cabinet

MDCK - MADIN-DARBY Canine Kidney cells

BT – Back Titration

RBC – Red Blood Cells

TRBCs – Turkey Red Blood Cells

GPRBCs – Guinea Pig Red Blood Cells

NP - Nucleoprotein

HRP – horseradish peroxidase

RT – room temperature

TMB -3, 3’, 5, 5’-Tetramethylbenzidine

OD-optical densit7
